# Double DQN-based secrecy energy efficiency and fairness performance in IRS-assisted NOMA systems with friendly jamming

**DOI:** 10.1371/journal.pone.0352324

**Published:** 2026-07-01

**Authors:** Hong Nguyen-Thi, Ngan Chu-Thi, Sang Quang-Nguyen, Dung Tran-Tien, Anh Le-Thi

**Affiliations:** 1 Posts and Telecommunications Institute of Technology, Hanoi, Vietnam; 2 School of Information and Communication, Hanoi University of Industry, Hanoi, Vietnam; 3 Posts and Telecommunications Institute of Technology, Ho Chi Minh City, Vietnam; Shenzhen University, CHINA

## Abstract

Faced with the challenge of increasing energy consumption and the need for sustainability in 6th generation (6G) wireless communication networks, this paper investigates the secrecy energy efficiency (SEE) and user fairness performance in an intelligent reflecting surface (IRS)-assisted non-orthogonal multiple access (NOMA) system in the presence of a friendly jammer and a passive eavesdropper over THz-Rician channels. We formulate both optimization objectives: maximizing the system’s total SEE and maximizing the maximum-min SEE to guarantee fairness for the worst-case user. Additionally, Jain’s fairness index is used to quantitatively evaluate the SEE balance among users. To solve these problems, we apply a Double Deep Q-Network (Double DQN)-based SEE of our proposed system model to jointly optimize power allocation and IRS phase shifts. This proposed approach enables efficient learning of optimal policies in dynamic environments without requiring explicit knowledge of the channel distribution. Furthermore, conventional Deep Q-Netowrk (DQN) and random allocation strategies are also implemented for comparison with Double DQN. Simulation results are presented for both IRS-assisted NOMA and OMA (orthogonal multiple access) systems to highlight the advantages of NOMA in terms of the secrecy-energy trade-off and spectral efficiency. Finally, the effects of system essential parameters, such as transmitted power, the number of IRS elements, atmospheric absorption coefficients, and the passive eavesdropper’s position, are examined. These simulation results show that the proposed Double DQN-based scheme significantly outperforms DQN and random methods in both SEE maximization and fairness enhancement, and that the NOMA system outperforms the OMA system. These findings confirm that the proposed model provides a basis for deploying a secure, energy-efficient, and sustainable wireless communication for future 6G networks.

## 1 Introduction

The rapid increase in large-scale connectivity and digital transformation services in 6G networks is posing serious challenges to global energy consumption [1,2]. Information and Communication Technology (ICT) infrastructure is a major energy consumer and contributes significantly to greenhouse gas emissions worldwide. According to research reports on environmental impact [3], the ICT sector accounts for approximately 3–4% of total global carbon emissions, and this figure is projected to continue to increase sharply due to the explosion of applications such as the Internet of Things (IoT), artificial intelligence, and big data applications. In particular, with next-generation wireless networks deploying dense base stations, using high-frequency bands, and increasingly complex signal processing, system energy consumption can increase exponentially if appropriate optimal solutions and policies are not in place.

In this context, building the sustainability of future network infrastructure needs to be based on research into green communication systems. This is not only a potential research direction but has become an essential requirement towards 6G networks [4]. In the design and operation of 6G systems, solutions to minimize energy consumption, optimize network resources, and limit carbon emissions are considered core measures. Therefore, integrating advanced energy-saving techniques across the physical and resource management layers is a crucial approach to balancing system performance and environmental impact.

Numerous studies and proposals have been proposed to meet the stringent energy-efficiency requirements of 6G networks. The increasing complexity of 6G networks to meet ever-evolving communication requirements leads to network designs with architectural features such as data-signal separation and dynamic resource allocation. This further highlights the need for energy-efficient, intelligent optimization mechanisms [3,4]. Consequently, many new physical-layer technologies have been proposed towards green and sustainable communication systems. Among them, Intelligent Reflecting Surface (IRS) has attracted considerable attention in the research community and is listed as one of the key technologies in beyond 5G (B5G) networks [5,6]. The IRS can reconfigure the propagation environment by using almost passive energy-consuming reflective elements. By flexibly adjusting the phase shifts of these elements, incident and reflected signals can be adjusted to produce a higher-quality resonant signal for legitimate users, reduce interference, and limit signal leakage into unwanted areas. Studies on IRS have recently been reported for both terrestrial and satellite communication systems, demonstrating its effectiveness in enhancing transmission reliability, coverage, and secrecy performance under different propagation conditions [7,8]. Compared to traditional active techniques, such as relay stations, multi-input multi-output (MIMO) technologies, and active beamforming, this technology does not use radio frequency (RF) chains to amplify the signal, making it relatively easy to integrate into networks and a promising low-power solution for power-constrained wireless systems. Therefore, the IRS contributes to improving the system’s spectral and energy efficiencies, enhancing its security against eavesdroppers [4,5], and reducing the carbon footprint of next-generation wireless networks [9].

Along with the IRS, Non-Orthogonal Multiple Access (NOMA) is recognized as an effective advanced access technique for supporting high-volume connectivity in beyond 5G and 6G networks [10]. Simply put, NOMA works by having multiple users share the same time-frequency resources via a power-domain NOMA (PD-NOMA) [11–13]. By efficiently utilizing these resources, NOMA improves spectrum utilization efficiency and user fairness while reducing transmitter power. This is an advantage of this technology over traditional orthogonal multiple access (OMA). Extensive research on NOMA has been reported in recent years, addressing a wide range of scenarios, including perfect/imperfect CSI and multi-hop communications. These studies further demonstrate the flexibility and applicability of NOMA in next-generation wireless networks [14,15]. Therefore, combining IRS and NOMA yields a robust solution that improves system performance metrics while reducing overall energy consumption [16–19]. In particular, IRS-aided NOMA systems can simultaneously leverage the benefits of passive beamforming and power-domain multiplexing. This offers a promising approach for green, energy-efficient 6G communication systems [20].

Besides energy-efficiency requirements, information security is a significant challenge in wireless communication networks, in general, and in 6G networks in particular, due to the open nature of the wireless environment [21]. Traditional security methods based on high-level cryptography – layers above the physical layer – often require high computational costs. This is unsuitable for large-scale IoT networks in the future because network devices have limited configuration options. As a result, physical layer security (PLS) has emerged as an effective solution. Unlike complex traditional cryptography methods, PLS exploits the randomness of the transmission channel to ensure system security. Specifically, jamming-friendly methods and artificial noise (AN) are two key approaches in this solution [22].

However, enhancing system security parameters using AN devices, jammers, beamforming, or cooperative relay transmission techniques often increases system power consumption. As a result, there is a trade-off between system security parameters and energy efficiency. This is even more significant given the stringent sustainability requirements in next-generation radio systems. To assess this trade-off, the proposed Security Energy Efficiency Index (SEE) is defined as the ratio of the security data rate to total power consumption [23]. Therefore, the optimal SEE in 6G and beyond not only enhances security but also ensures efficient energy use. However, when advanced wireless technologies are integrated, the system often becomes a non-convex problem and is difficult to solve using conventional mathematical methods. Thus, an effective, real-time optimization framework that achieves optimal SEE remains an open and challenging research problem [24,25].

Although many studies have focused on optimizing the performance of IRS-aided NOMA systems under security constraints, most current works primarily aim to maximize security rate or total system throughput, while inadequately addressing the energy-efficiency problem of security in the context of 6G green networks [20,26,27]. Some studies [28–30] have examined the energy efficiency index; however, they often rely on idealized assumptions, such as a perfect channel state or a static transmission environment [31,32]. Therefore, these findings are limited in their applicability to highly volatile real-world scenarios.

In addition, traditional optimization methods, such as alternating optimization (AO) [33], successive convex approximation (SCA) [34], or algorithms based on mathematical programming [35] often face high computational complexity and are difficult to scale as the system size increases [36]. Especially in IRS systems with a large number of elements, simultaneously optimizing power allocation for legitimate users and adjusting the phase shift of element reflections makes the problem complex and difficult to solve in real time. Furthermore, these optimization methods often require complete and accurate channel information, while the transmission environment of 6G networks is dynamic and random. As a result, these methods are not feasible for next-generation wireless network systems.

To overcome the above limitations, reinforcement learning (RL), especially deep learning methods such as Deep Q-Learning (DQL), has emerged as a promising tool in solving resource optimization problems in dynamic environments [37]. RL is based on learning optimal policies through interaction with the environment, without requiring accurate modeling of channel characteristics. Applying RL within a system enables it to adapt flexibly to changes in the transmission environment and significantly reduces computational complexity in practical implementations [38].

Based on the above observations, the authors are motivated to investigate and apply deep reinforcement learning techniques to the secrecy-energy-efficiency (SEE) performance optimization problem for the proposed IRS-aided PD-NOMA system, aiming to address the complex non-convexity and meet the real-time adaptation requirements of future green and smart wireless networks.

The main contributions of this paper can be summarized as follows:

- First, we propose an IRS-assisted NOMA communication framework with a friendly jammer in the THz band, accounting for both direct and IRS-reflected links under realistic channel conditions, including molecular absorption and Rician fading.
- Second, we formulate the secrecy energy efficiency (SEE) optimization problem with two objectives: maximizing total SEE and maximizing the minimum SEE, to enhance system efficiency and user fairness, respectively. This problem shows the trade-off between security and energy consumption in green wireless communication systems.
- Third, a Double Deep Q-learning-based framework is developed by modeling the problem as a Markov Decision Process (MDP), where both power allocation coefficients and IRS phase shifts are jointly optimized. In particular, Double DQN is employed to improve learning stability and convergence while requiring no explicit channel state information. Moreover, we design a constraint-aware reward mechanism that incorporates SINR and secrecy capacity requirements, enabling the learning agent to balance performance maximization and feasibility during training.
- Fourth, simulation results are implemented for our proposed Double DQN-based scheme and benchmark, including the DQN-based scheme and random policy, to demonstrate the advantages of Double DQN. Additionally, the IRS-assisted OMA scheme is considered to highlight our proposed IRS-NOMA performance in terms of SEE and user fairness.
- Finally, numerical analyses provide key insights into the impact of system parameters, such as the number of IRS elements, transmission power, and channel characteristics, highlighting their critical roles in achieving energy-efficient and secure communications.

The remainder of the paper is organized as follows. Section II presents the proposed IRS-assisted PD-NOMA system model and derives SINRs and system security capacity (SC) formulas. Section III builds the objective function problem with SEE formula cases. Section IV proposes a DQN-based solution method, modeling the problem as a Markov decision process and designing a Double DQN algorithm for SEE optimization. Section V presents simulation results and performance evaluations demonstrating the effectiveness of the proposed method across different system configurations and parameter settings. Finally, Section VI concludes the paper on the achieved results and affirms the role of the proposed system as a potential application in future green networks.

## 2 Proposed NOMA-assisted IRS system model and signal transmission

### 2.1 Proposed NOMA-assisted IRS system model

**Fig 1:**
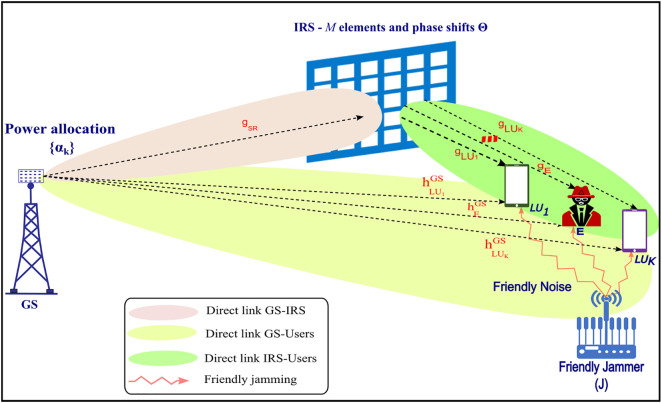
Proposed IRS-assisted PD-NOMA system model with friendly jamming.

In this paper, we consider a downlink power-domain non-orthogonal multiple access (PD-NOMA) system assisted by a reflecting, reconfigurable intelligent surface (IRS), as illustrated in Fig 1, operating over Rician THz channels. The system consists of one ground base station (GS), one IRS with *M* passive elements, *K* legitimate users (LUs), one eavesdropper (E), and a friendly jammer (J). In this model, the ground station (GS) communicates with the end users via both the direct links and the IRS-reflected links. The distances from the GS to the IRS, the *K* end users, and the eavesdropper are denoted by $d_{IRS}^{GS}$, $d_{k}^{GS}$ for k=1,…,K, and $d_{E}^{GS}$, respectively. Likewise, the distances from the IRS to the *n*-th user and the eavesdropper are denoted by $d_{k}^{IRS}$ and $d_{E}^{IRS}$, respectively. Moreover, the distances from friendly jammer J to $LU_{k}$ and Ev are denoted as $d_{k}^{J}$, and $d_{Ev}^{J}$. In our model, the friendly jammer *J* is assumed to be a cooperative and centrally coordinated node under the control of the ground station (GS). The GS has full knowledge of the jammer’s transmit power $Q_{J}$ and its location. The jammer operates in half-duplex mode, continuously transmitting a jamming signal to degrade the eavesdropper’s received signal. Finally, since *E* is a passive, non-cooperative entity, instantaneous CSI acquisition is practically infeasible. We adopt the statistical CSI assumption – the GS knows the eavesdropper’s location and the large-scale channel statistics.

The GS transmits a superimposed signal to all users based on the NOMA principle. Specifically, the transmitted signal is given by


$$\begin{array}{c} x_{GS}=\sum_{k=1}^{K}\sqrt{\alpha_{k}}x_{k}, \end{array}$$ (1)

where $x_{k}$ denotes the information signal intended for the *k*-th user, and $\alpha_{k}$ is the corresponding power allocation coefficient that satisfies $\sum_{k=1}^{K}\alpha_{k}=1$. And, without loss of generality, in this model, we assume that legitimate user 1 (*LU*_1_) is the user closest to the GBS and the IRS, followed by user 2 (*LU*_2_), and so on, with the $K^{th}$ user being the farthest, then $\alpha_{k+1}>\alpha_{k}$ with *k* = 1,...,*K* to ensure that users with poorer channel conditions are allocated more power.

All wireless channels are modeled as an independent Rician fading channels, especially, *g* denotes IRS-related channels while *h* denotes direct links. Then each Rician channel $ch_{R}$ is generally expressed as


$$\begin{array}{c} ch_{R}=\frac{1}{\sqrt{L_{R}(d)}}\left(\sqrt{\frac{K_{R}}{K_{R}+1}}c{h_{R}}^{\text{LoS}}+\sqrt{\frac{1}{K_{R}+1}}c{h_{R}}^{\text{NLoS}}\right), \end{array}$$ (2)

with $ch_{R}\in \left\{g_{SR},g_{LU_{1\to K}},g_{E},h_{LU_{1\to K}}^{GS},h_{E}^{GS}\right\}$ and R∈{SR,1,...,K,E}.

Where $K_{R}$ is the Rician factor, $L_{R}(d)$ denotes the large-scale path loss. In the considered THz communication system, the path loss is modeled by incorporating both the free-space spreading loss and the molecular absorption loss. The path loss at distance *d* is expressed as [39]


$$\begin{array}{c} L_{R}(d)={\left(\frac{4\pi df}{c}\right)}^{2}e^{k_{f}d}, \end{array}$$ (3)

where *f* denotes the carrier frequency in the THz band, *d* is the transmission distance, *c* is the speed of light, and $k_{f}$ represents the frequency-dependent molecular absorption coefficient of the propagation medium. This coefficient depends on frequency, humidity, atmospheric pressure, and temperature [39,40]. And ${ch_{R}}^{LoS}$ is the deterministic line-of-sight (LoS) component, and ${ch_{R}}^{NLoS}\sim CN(0,1)$ is the non-line-of-sight (NLoS) component. In our paper, the channel vector from the ground base station (GS) to the IRS is represented by ${𝐠}_{SR}\in {\mathbb{C}}^{M\times 1}$, where *M* is the number of reflecting elements at the IRS, while channel vector from the IRS to LUs and eavesdropper are denoted by ${𝐠}_{LU_{k}}^{H}$ and ${𝐠}_{E}^{H}$, respectively, in ${\mathbb{C}}^{1\times M}$. In addition, the direct channel from the GS to the LUs and eavesdropper are denoted by $h_{LU_{k}}^{GS}$ and $h_{E}^{GS}$. All these links are assumed to experience independent Rician fading, following the Rician channel model given in (2), which consists of a deterministic line-of-sight (LoS) component and a random non-line-of-sight (NLoS) component weighted by the Rician factor.

Let Θ denote the coefficient matrix, respectively, defined as


$$\begin{array}{c} \Theta_{IRS}=diag\left(\sqrt{\beta_{IRS,1}}e^{j\theta_{IRS,1}},\ldots ,\sqrt{\beta_{IRS,M}}e^{j\theta_{IRS,M}}\right), \end{array}$$ (4)

where $\beta_{IRS,m}$ represents the amplitude coefficients of the *m*-th element, respectively, satisfying $\beta_{IRS,m}\leq 1,\;\forall m,$ and $\theta_{IRS,m}\in [0,2\pi )$ denotes the corresponding phase shifts. For clarity, the main notations used in the NOMA–IRS system model are summarized in Table 1.

**Table 1:** Notation for NOMA–IRS System Model.

Symbol	Description
*K*	Number of legitimate users (LUs)
*M*	Number of reflecting elements at the IRS
*k*	Index of user, k=1,…,K
*m*	Index of IRS element, m=1,…,M
$\alpha_{k}$	Power allocation coefficient for user *k*
α	Power allocation vector $\left\{\alpha_{1},\ldots ,\alpha_{K}\right\}$
$\theta_{m}$	Phase shift of the *m*-th IRS element
Θ	IRS phase shift matrix
$\Theta_{IRS}$	IRS reflection coefficient matrix
$Q_{GS}$	Transmit power of the ground station (GS)
$Q_{J}$	Transmit power of the friendly jammer
$x_{k}$	Information signal intended for user *k*
$h_{k}^{GS}$	Channel coefficient from GS to user *k*
**g**_SR_	Channel vector from GS to IRS
${𝐠}_{{LU}_{k}}$	Channel vector from IRS to user *k*
${𝐠}_{E}$	Channel vector from IRS to eavesdropper
$\gamma_{k}^{{LU}_{k}}$	SINR at user *k*
$\gamma_{k}^{th}$	Minimum SINR threshold
$SC_{k}^{IRS}$	Secrecy capacity of user *k*
$SC_{0}^{th}$	Minimum secrecy capacity requirement
$SEE_{k}$	Secrecy energy efficiency of user *k*
SEE_total_	Total secrecy energy efficiency
$P_{total}$	Total power consumption

### 2.2 Signal transmission

The received signal at the $k^{th}$ legitimate user consists of the superposition of the IRS-assisted link ($y_{k}^{IRS}$), the direct GS link ($y_{k}^{GS}$), and the jammer signal from J ($y_{k}^{J}$) expressed as


$$\begin{array}{c} y_{{LU}_{k}}=y_{k}^{IRS}+y_{k}^{GS}+y_{k}^{J}+n_{{LU}_{k}}. \end{array}$$ (5)

Here, $n_{k}\sim CN(0,\sigma^{2})$ is an additive white Gaussian noise at $LU_{k}$; the IRS-assisted signal is given by


$$\begin{array}{cc} y_{k}^{IRS} & ={𝐠}_{{LU}_{k}}^{H}\Theta {𝐠}_{SR}\sqrt{Q_{GS}}x_{GS}\\ & ={𝐠}_{{LU}_{k}}^{H}\Theta {𝐠}_{SR}\sqrt{Q_{GS}}\sum_{k=1}^{K}\sqrt{\alpha_{k}}x_{k}, \end{array}$$ (6)

where $Q_{GS}$ is the transmit power of the GS. The signal component of direct GS link $y_{k}^{GS}$ can be expressed as


$$\begin{array}{c} y_{k}^{GS}=h_{k}^{GS}\sqrt{Q_{GS}}\sum_{k=1}^{N}\sqrt{\alpha_{k}}x_{k}. \end{array}$$ (7)

And $y_{k}^{J}$ with $Q_{J}$ is the jamming power and $x_{J}$ is the jamming signal, can be given by


$$\begin{array}{c} y_{k}^{J}=h_{k}^{J}\sqrt{Q_{J}}x_{J}. \end{array}$$ (8)

Similarly, the received signal at the eavesdropper is expressed as


$$\begin{array}{c} y_{Ev}={𝐠}_{E}^{H}\Theta {𝐠}_{SR}\sqrt{Q_{GS}}\sum_{k=1}^{K}\sqrt{\alpha_{k}}x_{k}+h_{E}^{GS}\sqrt{Q_{GS}}\sum_{k=1}^{K}\sqrt{\alpha_{k}}x_{k}+h_{E}^{J}\sqrt{Q_{J}}x_{J}+n_{E}. \end{array}$$ (9)

Here, $n_{E}\sim CN(0,\sigma^{2})$ is an additive white Gaussian noise at Ev. And, the channels from the friendly jammer *J* to the legitimate user and the eavesdropper are denoted by $h_{LU_{k}}^{J}$ and $h_{E}^{J}$, respectively. It is assumed that no propagation link exists between the jammer and the IRS. Therefore, the jamming signal is only received through the direct links at the legitimate user and the eavesdropper. Both $h_{LU_{k}}^{J}$ and $h_{E}^{J}$ are assumed to follow independent Rician fading channels as described in (2).

### 2.3 SINRS and SC formulations

At the *k*-th user, in order to successfully decode its own signal $x_{k}$, the legitimate user ${LU}_{k}$ is required to first decode and remove the signals of users with higher power allocation (i.e., users k+1,…,K) by employing successive interference cancellation (SIC). Without loss of generality, the users are ordered based on their channel conditions, where LU_1_ is the nearest user and ${LU}_{K}$ is the farthest user. It is assumed that the legitimate users are capable of estimating and mitigating the effect of the jamming signal; therefore, the interference caused by the jammer is negligible at the legitimate users. Accordingly, the received signal-to-interference-plus-noise ratio (SINR) at ${LU}_{k}$ for decoding the signal of user *K* is given by


$$\begin{array}{c} \gamma_{K}^{LU_{k}}=\frac{\alpha_{K}Q_{GS}{\left\|h_{k}^{GS}+g_{LU_{k}}^{H}\Theta g_{SR}\right\|}^{2}}{Q_{GS}\sum_{i=1}^{K-1}\alpha_{i}{\left\|h_{k}^{GS}+g_{LU_{k}}^{H}\Theta g_{SR}\right\|}^{2}+\delta_{LU_{k}}^{2}}. \end{array}$$ (10)

Similarly, the SINR for decoding the signal of user K−1 at $LU_{k}$ is expressed as


$$\begin{array}{c} \gamma_{K-1}^{LU_{k}}=\frac{\alpha_{K-1}Q_{GS}{\left\|h_{k}^{GS}+g_{LU_{k}}^{H}\Theta g_{SR}\right\|}^{2}}{Q_{GS}\left(\sum_{i=1}^{K-1}\alpha_{i}+\xi \alpha_{K}\right){\left\|h_{k}^{GS}+g_{LU_{k}}^{H}\Theta g_{SR}\right\|}^{2}+\delta_{LU_{k}}^{2}}. \end{array}$$ (11)

In general, the SINR at ${LU}_{k}$ for decoding the signal $x_{k}$ at $LU_{k}$ is given by


$$\begin{array}{c} \gamma_{k}^{LU_{k}}=\frac{\alpha_{k}Q_{GS}{\left\|h_{k}^{GS}+g_{LU_{k}}^{H}\Theta g_{SR}\right\|}^{2}}{Q_{GS}\left(\sum_{j=1}^{k-1}\alpha_{j}+\xi \sum_{j=k+1}^{K}\alpha_{j}\right){\left\|h_{k}^{GS}+g_{LU_{k}}^{H}\Theta g_{SR}\right\|}^{2}+\delta_{LU_{k}}^{2}}. \end{array}$$ (12)

For illustration, the SINRs corresponding to LU_2_ and LU_1_ at $LU_{k}$ are respectively given by


$$\begin{array}{c} \gamma_{2}^{LU_{k}}=\frac{\alpha_{2}Q_{GS}{\left\|h_{k}^{GS}+g_{LU_{k}}^{H}\Theta g_{SR}\right\|}^{2}}{Q_{GS}\left(\alpha_{1}+\xi \sum_{j=3}^{K}\alpha_{j}\right){\left\|h_{k}^{GS}+g_{LU_{k}}^{H}\Theta g_{SR}\right\|}^{2}+\delta_{LU_{k}}^{2}}. \end{array}$$ (13)

$$\begin{array}{c} \gamma_{1}^{{LU}_{k}}=\frac{\alpha_{1}Q_{GS}{\left\|h_{k}^{GS}+{𝐠}_{{LU}_{k}}^{H}\Theta {𝐠}_{SR}\right\|}^{2}}{Q_{GS}\left(\xi \sum_{j=2}^{K}\alpha_{j}\right){\left\|h_{k}^{GS}+{𝐠}_{{LU}_{k}}^{H}\Theta {𝐠}_{SR}\right\|}^{2}+\delta_{{LU}_{k}}^{2}}. \end{array}$$ (14)

Here, ξ∈[0,1] denotes the residual interference factor caused by imperfect successive interference cancellation (SIC). Specifically, ξ=0 corresponds to perfect SIC, while ξ>0 reflects the presence of residual interference due to decoding errors or hardware limitations, leading to incomplete cancellation of previously decoded signals. Moreover, $\delta_{{LU}_{k}}^{2}$ represents the variance of the additive white Gaussian noise (AWGN) at the *k*-th legitimate user, where the noise is modeled as a circularly symmetric complex Gaussian random variable with distribution $n_{{LU}_{k}}\sim CN(0,\delta_{{LU}_{k}}^{2})$.

At the eavesdropper *E*, the SINR for intercepting the signal intended for the *k*-th legitimate user is expressed as


$$\begin{array}{c} \gamma_{k}^{E}=\frac{\alpha_{k}Q_{GS}{\left\|h_{E}^{GS}+g_{E}^{H}\Theta g_{SR}\right\|}^{2}}{Q_{GS}\left(\sum_{j=1}^{k-1}\alpha_{j}+\xi \sum_{j=k+1}^{K}\alpha_{j}\right){\left\|h_{E}^{GS}+g_{E}^{H}\Theta g_{SR}\right\|}^{2}+Q_{J}{\left\|h_{E}^{J}\right\|}^{2}+\delta_{E}^{2}}. \end{array}$$ (15)

Here, $h_{E}^{GS}$ denotes the direct channel from the GS to the eavesdropper, and $\delta_{E}^{2}$ is the variance of the additive white Gaussian noise (AWGN) at *E*.

According to Shannon’s theorem, the achievable channel capacity is given by


$$\begin{array}{c} C=B\log_{2}(1+SINR)\;\text{(bps)}, \end{array}$$ (16)

where *B* denotes the system bandwidth.

For the farthest legitimate user ${LU}_{K}$, no prior SIC operation is required. Therefore, its achievable rate is expressed as


$$\begin{array}{c} C_{{LU}_{K}}^{\left[K\right]}=B\log_{2}\left(1+\gamma_{K}^{{LU}_{K}}\right). \end{array}$$ (17)

For the $(K-1{)}^{th}$ legitimate user, the signal of user *K* must first be successfully decoded before detecting its own signal. Hence, the achievable rate of ${LU}_{K-1}$ is written as


$$\begin{array}{c} C_{{LU}_{K-1}}^{\left[K-1\right]}=\left\{ \begin{array}{cc} B\log_{2}\left(1+\gamma_{K-1}^{{LU}_{K-1}}\right), & \text{if }\gamma_{K}^{{LU}_{K-1}}\geq \gamma_{K}^{th},\\ 0, & \text{otherwise}, \end{array}\right. \end{array}$$ (18)

where $\gamma_{K}^{th}$ is the minimum SINR threshold required for successfully decoding the signal of user *K*.

In general, for the *k*-th legitimate user ${LU}_{k}$, successful decoding of its own signal requires that the signals of users k+1,k+2,…,K be correctly decoded in advance via SIC. Thus, the achievable rate of ${LU}_{k}$ is given by


$$\begin{array}{c} C_{{LU}_{k}}^{\left[k\right]}=\left\{ \begin{array}{cc} B\log_{2}\left(1+\gamma_{k}^{{LU}_{k}}\right), & \text{if }\gamma_{n}^{{LU}_{k}}\geq \gamma_{n}^{th},\;\forall n\in \{k+1,\ldots ,K\},\\ 0, & \text{otherwise}. \end{array}\right. \end{array}$$ (19)

Similarly, for LU_2_ and LU_1_, their achievable rates can be expressed as


$$\begin{array}{c} C_{{LU}_{2}}^{\left[2\right]}=\left\{ \begin{array}{cc} B\log_{2}\left(1+\gamma_{2}^{{LU}_{2}}\right), & \text{if }\gamma_{n}^{{LU}_{2}}\geq \gamma_{n}^{th},\;\forall n\in \{3,\ldots ,K\},\\ 0, & \text{otherwise}, \end{array}\right. \end{array}$$ (20)

and


$$\begin{array}{c} C_{{LU}_{1}}^{\left[1\right]}=\left\{ \begin{array}{cc} B\log_{2}\left(1+\gamma_{1}^{{LU}_{1}}\right), & \text{if }\gamma_{n}^{{LU}_{1}}\geq \gamma_{n}^{th},\;\forall n\in \{2,\ldots ,K\},\\ 0, & \text{otherwise}. \end{array}\right. \end{array}$$ (21)

At the eavesdropper *E*, the achievable rate for intercepting the signal intended for user *K* is given by


$$\begin{array}{c} C_{E}^{\left[K\right]}=B\log_{2}\left(1+\gamma_{K}^{E}\right). \end{array}$$ (22)

For a general $LU_{k}$ signal, the eavesdropper must also successfully decode the higher-power signals corresponding to users k+1,…,K before attempting to decode the $k^{th}$ signal. Accordingly, the achievable interception rate at *E* is expressed as


$$\begin{array}{c} C_{E}^{\left[k\right]}=\left\{ \begin{array}{cc} B\log_{2}\left(1+\gamma_{k}^{E}\right), & \text{if }\gamma_{n}^{E}\geq \gamma_{n}^{th},\;\forall n\in \{k+1,\ldots ,K\},\\ 0, & \text{otherwise}. \end{array}\right. \end{array}$$ (23)

In particular, for the signal intended for *LU*_1_, the achievable interception rate at the eavesdropper is given by


$$\begin{array}{c} C_{E}^{\left[1\right]}=\left\{ \begin{array}{cc} B\log_{2}\left(1+\gamma_{1}^{E}\right), & \text{if }\gamma_{n}^{E}\geq \gamma_{n}^{th},\;\forall n\in \{2,\ldots ,K\},\\ 0, & \text{otherwise}. \end{array}\right. \end{array}$$ (24)

Based on the above achievable rates, the secrecy capacity (SC) of the $k^{th}$ legitimate user is defined as


$$\begin{array}{c} SC_{k}^{IRS}={\left[C_{{LU}_{k}}^{\left[k\right]}-C_{E}^{\left[k\right]}\right]}^{+}, \end{array}$$ (25)

where $\left[x{]}^{+}=\max(x,0\right)$.

Equivalently, the secrecy capacity can be written as


$$\begin{array}{c} SC_{k}^{IRS}=\left\{ \begin{array}{cc} B\log_{2}\left(\frac{1+\gamma_{k}^{{LU}_{k}}}{1+\gamma_{k}^{E}}\right), & \text{if }\gamma_{n}^{{LU}_{k}}\geq \gamma_{n}^{th}\text{ and }\gamma_{n}^{E}\geq \gamma_{n}^{th},\;\forall n\in \{k+1,\ldots ,K\},\\ 0, & \text{otherwise}. \end{array}\right. \end{array}$$ (26)

By substituting Eq. 12 and Eq. 15 into Eq. 26, we can express $SC_{k}^{IRS}$ as follows


$$\begin{array}{c} SC_{k}^{IRS}=B{\left[\log_{2}\frac{1+\frac{\alpha_{k}Q_{GS}{\left\|h_{k}^{GS}+g_{LU_{k}}^{H}\Theta g_{SR}\right\|}^{2}}{Q_{GS}\left(\sum_{j=1}^{k-1}\alpha_{j}+\xi \sum_{j=k+1}^{K}\alpha_{j}\right){\left\|h_{k}^{GS}+g_{LU_{k}}^{H}\Theta g_{SR}\right\|}^{2}+\delta_{LU_{k}}^{2}}}{1+\frac{\alpha_{k}Q_{GS}{\left\|h_{E}^{GS}+g_{E}^{H}\Theta g_{SR}\right\|}^{2}}{Q_{GS}\left(\sum_{j=1}^{k-1}\alpha_{j}+\xi \sum_{j=k+1}^{K}\alpha_{j}\right){\left\|h_{E}^{GS}+g_{E}^{H}\Theta g_{SR}\right\|}^{2}+Q_{J}{\left\|h_{E}^{J}\right\|}^{2}+\delta_{E}^{2}}}\right]}^{+} \end{array}$$ (27)

## 3 Problem formulations

### 3.1 Power consumption model

To evaluate the secrecy energy efficiency (SEE) of the considered system, the total power consumption is modeled by accounting for both transmit power and circuit power components.

The total power consumption of the system is expressed as


$$\begin{array}{c} P_{total}=\frac{Q_{GS}}{\beta_{PAE}^{GS}}+P_{c}^{GS}+\sum_{k=1}^{K}P_{c}^{{LU}_{k}}+P_{c}^{IRS}+\frac{Q_{J}}{\beta_{PAE}^{jammer}}, \end{array}$$ (28)

where $Q_{GS}$ and $Q_{J}$ denote the transmit power at the ground station (GS) and the friendly jammer, respectively.

Here, $\beta_{PAE}^{BS}\in (0,1)$ and $\beta_{PAE}^{jammer}\in (0,1)$ represent the power amplifier efficiency (PAE) at the GS and the jammer, respectively. Typically, these values are set to constants, e.g., $\beta_{PAE}^{GS}=0.5$ and $\beta_{PAE}^{jammer}=0.4$.

The term $P_{c}^{GS}$ denotes the circuit power consumption at the GS. Similarly, $\sum_{k=1}^{K}P_{c}^{{LU}_{k}}$ represents the total circuit power consumption of all *K* legitimate users. For simplicity, the circuit power consumption at each user is assumed to be identical.

The power consumption of the IRS is modeled as


$$\begin{array}{c} P_{c}^{IRS}=MP_{element}^{IRS}+P_{controller}, \end{array}$$ (29)

where *M* is the number of reflecting elements at the IRS, $P_{element}^{IRS}$ denotes the power consumption of each passive element (typically in the range of 5–10 mW per element), and $P_{controller}$ is the power consumption of the IRS controller (typically 50–200 mW).

It is worth noting that, under the total transmit power constraint at the GS, the power allocation coefficients $\{\alpha_{k}{\}}_{k=1}^{K}$ satisfy $\sum_{k=1}^{K}\alpha_{k}=1$. Therefore, the total transmit power at the GS remains constant, and the optimization primarily depends on the allocation of $\left\{\alpha_{k}\right\}$ among users.

Based on the above power consumption model, the secrecy energy efficiency will be evaluated in the following section under two optimization criteria, namely the maximization of the total SEE and the max–min SEE among users.

### 3.2 Case 1: Maximization of total secrecy energy efficiency

The total secrecy energy efficiency (SEE) of the system is defined as the ratio between the sum secrecy capacity of all legitimate users and the total power consumption, which is expressed as


$$\begin{array}{c} {SEE}^{\left(1\right)}=\frac{\sum_{k=1}^{K}SC_{k}^{IRS}}{P_{total}}. \end{array}$$ (30)

By substituting the expression of the secrecy capacity, SEE^(1)^ can be rewritten as


$$\begin{array}{c} {SEE}^{\left(1\right)}=\frac{\sum_{k=1}^{K}{\left[B\log_{2}\left(\frac{1+\gamma_{k}^{{LU}_{k}}}{1+\gamma_{k}^{E}}\right)\right]}^{+}}{P_{total}}. \end{array}$$ (31)

Accordingly, the optimization problem is formulated as


$$\begin{array}{c} \max_{\Theta ,\{\alpha_{k}\}}\;{SEE}^{\left(1\right)} \end{array}$$ (32a)

$$\begin{array}{c} \text{s.t.}\;\alpha_{k+1}\geq \alpha_{k},\;\forall k=1,\ldots ,K-1, \end{array}$$ (32b)

$$\begin{array}{c} \sum_{k=1}^{K}\alpha_{k}=1, \end{array}$$ (32c)

$$\begin{array}{c} \theta_{m}\in [0,2\pi ),\;\forall m=1,\ldots ,M, \end{array}$$ (32d)

$$\begin{array}{c} \gamma_{k}^{{LU}_{k}}\geq \gamma_{k}^{th},\;\forall k, \end{array}$$ (32e)

$$\begin{array}{c} SC_{k}^{IRS}\geq SC_{0}^{th},\;\forall k. \end{array}$$ (32f)

Here, $\gamma_{k}^{th}$ and $SC_{0}^{th}$, are introduced to ensure the system’s quality-of-service (QoS) and security requirements in which $\gamma_{k}^{th}$ is the minimum SINR at user *k*, and $SC_{0}^{th}$ is the minimum required secrecy capacity. In particular, the constraint $\gamma_{k}^{{LU}_{k}}\geq \gamma_{k}^{th}$ ensures that the difference between the legitimate channel capacity and the eavesdropping channel capacity remains above a prescribed threshold.

**Proposition 1 (Feasible Region of Power Allocation).** *Under the SINR constraint in (32e) and the secrecy-capacity constraint in (32f), the feasible set of the power allocation coefficients* $\left\{\alpha_{k}\right\}$ *is implicitly constrained by coupled lower bounds.*

*Specifically, each* $\alpha_{k}$ *depends on the power allocation of other users as well as the channel conditions and IRS configuration. Consequently, the feasible region is highly coupled and non-convex, and cannot be decomposed into independent closed-form bounds for individual users.*

*This intrinsic coupling significantly complicates the optimization problem and motivates the use of learning-based approaches.*

*Proof:* The proof is provided in S1 Appendix A.

*Remark 1:* Although the above analysis yields explicit lower bounds on $\alpha_{k}$, these bounds are inherently coupled with other optimization variables and thus cannot be used to decouple the problem. As a result, the original constraints in (32e) and (32f) are preserved, while the derived expressions are mainly used to provide insights into the problem structure and guide the design of the proposed DRL-based solution.

### 3.3 Case 2: Max–min secrecy energy efficiency

To ensure fairness among users, the secrecy energy efficiency (SEE) is defined based on the worst-case user as


$$\begin{array}{c} {SEE}^{\left(2\right)}=\frac{\min_{k=1,\ldots ,K}\left\{SC_{k}^{IRS}\right\}}{P_{total}}. \end{array}$$ (33)

By substituting the secrecy capacity expression, SEE^(2)^ can be rewritten as


$$\begin{array}{c} {SEE}^{\left(2\right)}=\frac{\min_{k=1,\ldots ,K}\left\{{\left[B\log_{2}\left(\frac{1+\gamma_{k}^{{LU}_{k}}}{1+\gamma_{k}^{E}}\right)\right]}^{+}\right\}}{P_{total}}. \end{array}$$ (34)

Accordingly, the max–min SEE optimization problem is formulated as


$$\begin{array}{c} \max_{\Theta ,\{\alpha_{k}\}}\;{SEE}^{\left(2\right)} \end{array}$$ (35a)

$$\begin{array}{c} \text{s.t.}\;\alpha_{k+1}\geq \alpha_{k},\;\forall k=1,\ldots ,K-1, \end{array}$$ (35b)

$$\begin{array}{c} \sum_{k=1}^{K}\alpha_{k}=1, \end{array}$$ (35c)

$$\begin{array}{c} \theta_{m}\in [0,2\pi ),\;\forall m=1,\ldots ,M, \end{array}$$ (35d)

$$\begin{array}{c} \gamma_{k}^{{LU}_{k}}\geq \gamma_{k}^{th},\;\forall k, \end{array}$$ (35e)

$$\begin{array}{c} SC_{k}^{IRS}\geq SC_{0}^{th},\;\forall k. \end{array}$$ (35f)

*Remark 2*: The above formulation aims to maximize the minimum secrecy energy efficiency among all users, thereby guaranteeing fairness in the system. However, since the objective focuses on the worst-case user, the overall system efficiency may not be maximized.

To enable a fair comparison with Case 1, where the total SEE is directly optimized, we evaluate the total secrecy energy efficiency using the optimal solution obtained from the max–min SEE problem.

Let $\left\{\Theta^{*},\alpha_{k}^{*}\right\}$ denote the optimal solution to the above problem. The corresponding total secrecy energy efficiency is then computed as


$$\begin{array}{c} {SEE}_{total}^{\left(2\right)}=\frac{\sum_{k=1}^{K}SC_{k}^{IRS}(\Theta^{*},\{\alpha_{k}^{*}\})}{P_{total}(\Theta^{*},\{\alpha_{k}^{*}\})}. \end{array}$$ (36)

This evaluation ensures that both Case 1 and Case 2 are assessed under the same performance metric, thereby providing a fair and meaningful comparison between efficiency-oriented and fairness-oriented system designs.

*Remark 3*: Compared to the sum-SEE maximization, the max–min SEE problem introduces additional coupling due to the minimum operator across users. Together with the constraints in Proposition 1, the problem becomes highly non-convex and difficult to solve, which motivates the use of DRL-based approaches.

### 3.4 User fairness index

After analyzing the two objective function problems, Max-sum SEE and Max-Min SEE, in Subsections 3.3 and 3.4, this section proposes analyzing user fairness based on the achieved security energy efficiency.

To do this, Jain’s fairness index is used and defined as follows:


$$\begin{array}{c} {FI}_{SEE}=\frac{{\left(\sum_{k=1}^{K}SEE_{k}\right)}^{2}}{K\sum_{k=1}^{K}SEE_{k}^{2}}, \end{array}$$ (37)

here, $SEE_{k}=\frac{SC_{k}^{IRS}}{P_{total}}$ is the secrecy energy efficiency of the $k^{th}$ user.

The fairness index is calculated using the results obtained after optimizing the problems in subsections 3.2 and 3.3, i.e., $\Theta^{*},{\alpha_{k}}^{*}\}$. This index is used to compare the fair performance of the total SEE maximization scheme and the maximum-minimum SEE scheme. A value of FI_SEE_ leaning towards 1 indicates fairness among users in the system, meaning users achieve approximately the same SEE performance. Conversely, when the fairness index is small and leans towards 0.5, it indicates that the system is unbalanced or that there is a large difference in users’ SEE. This index allows for a quantitative assessment of the trade-off between system efficiency and user fairness across different optimization strategies, as further illustrated in the results section.

## 4 Double DQN-based SEE optimization for IRS-assisted NOMA systems

In this section, we formulate the SEE optimization problems in subsections 3.2 and 3.3 by a Markov Decision Process (MDP) framework. Due to the high non-convexity and the presence of constraint system variables, obtaining a globally optimal solution using conventional optimization techniques is computationally challenging. To address this, Deep Q-Network (DQN) and Double Deep Q-Network (Double DQN) algorithms are employed to develop efficient resource allocation strategies for the IRS-assisted NOMA system. The main objective is to maximize the system’s SEE by jointly optimizing power allocation, IRS phase shifts, and transmit power. These deep reinforcement learning (DRL) methods enable the system to make adaptive decisions in dynamic environments without requiring prior knowledge of channel distributions.

Additionally, the SEE optimization problems in Case 1 and Case 2 are addressed using a unified DRL framework. Both problems are modeled as MDPs with identical state and action spaces, while distinct reward functions are designed to capture their respective optimization objectives.

### 4.1 MDP formulation for SEE optimization

The SEE optimization problem is formulated as a Markov Decision Process (MDP), defined by the tuple (𝒮,𝒜,𝒫,ℛ,γ), where 𝒮, 𝒜, 𝒫, ℛ, and γ denote the state space, action space, state transition probability, reward function, and discount factor, respectively.

#### 4.1.1 State space.

At time step *t*, the state captures the estimated channel-related state information (CSI) and system configuration available at the GBS, which is defined as


$$\begin{array}{c} s_{t}=\left\{g_{SR},g_{LU_{k}},g_{E},h_{k}^{GS},h_{E}^{GS},h_{J-E},\alpha ,\Theta ,\gamma_{k}^{LU_{k}},\gamma_{k}^{E},P_{total}\right\}. \end{array}$$ (38)

Here, ${𝐠}_{SR}\in {\mathbb{C}}^{M\times 1}$ denotes the channel from the GS to the IRS, ${𝐠}_{LU_{k}}^{H}\in {\mathbb{C}}^{1\times M}$ and ${𝐠}_{E}^{H}\in {\mathbb{C}}^{1\times M}$ represent the IRS-to-user and IRS-to-eavesdropper channels, respectively. The terms $h_{k}^{GS}$ and $h_{E}^{GS}$ denote the direct links from the GS to the legitimate user and the eavesdropper, respectively, while $h_{J-E}$ represents the jammer-to-eavesdropper channel. The CSI variables in the state space are assumed to be obtained through practical pilot-assisted channel estimation, received signal measurements, and SINR feedback mechanisms available at the BS. Therefore, the proposed framework does not require perfect global CSI for iterative optimization. The vectors $\alpha =[\alpha_{1},\ldots ,\alpha_{K}]$ and $\Theta =diag(e^{j\theta_{1}},\ldots ,e^{j\theta_{M}})$ denote the power allocation coefficients and IRS phase shifts, respectively. The SINRs at the legitimate user and the eavesdropper are denoted by $\gamma_{k}^{{LU}_{k}}$ and $\gamma_{k}^{E}$, respectively, and $P_{total}$ is the total power consumption defined in Section 3.

#### 4.1.2 Action space.

The action at time step *t* corresponds to updating the system control variables, which is given by


$$\begin{array}{c} a_{t}=\left\{\Delta \alpha_{1},\ldots ,\Delta \alpha_{K},\Delta \theta_{1},\ldots ,\Delta \theta_{M},\Delta Q_{GS},\Delta Q_{J}\right\}. \end{array}$$ (39)

Here, $\Delta \alpha_{k}$ represents the adjustment of the power allocation coefficient for user *k*, $\Delta \theta_{m}$ denotes the phase shift adjustment of the *m*-th IRS element, and $\Delta Q_{GS}$ and $\Delta Q_{J}$ denote the transmit power adjustments at the GS and the jammer, respectively. These actions are discretized into finite levels to enable the application of DQN-based algorithms.

#### 4.1.3 Reward function.

The reward function is designed to reflect the SEE optimization objective while enforcing the system constraints. To penalize constraint violations, two binary variables are introduced as


$$\begin{array}{c} \eta_{k}^{\left(1\right)}=\left\{ \begin{array}{cc} 1, & \gamma_{k}^{{LU}_{k}}<\gamma_{k}^{th}\\ 0, & \text{otherwise} \end{array}\right. \end{array}$$ (40)

$$\begin{array}{c} \eta_{k}^{\left(2\right)}=\left\{ \begin{array}{cc} 1, & SC_{k}^{IRS}<SC_{0}^{th}\\ 0, & \text{otherwise} \end{array}\right. \end{array}$$ (41)

where $\eta_{k}^{\left(1\right)}$ and $\eta_{k}^{\left(2\right)}$ indicate violations of the SINR and secrecy capacity constraints, respectively.

In the problem of Total SEE Maximization, the reward is defined as


$$\begin{array}{c} r_{t}^{\left(1\right)}={SEE}_{total}-\lambda_{1}\sum_{k=1}^{K}\eta_{k}^{\left(1\right)}-\lambda_{2}\sum_{k=1}^{K}\eta_{k}^{\left(2\right)}. \end{array}$$ (42)

In the case of Max–Min SEE problem, the reward is defined based on the worst-case user as


$$\begin{array}{c} r_{t}^{\left(2\right)}=\min_{k=1,\ldots ,K}\{SEE_{k}\}-\lambda_{1}\sum_{k=1}^{K}\eta_{k}^{\left(1\right)}-\lambda_{2}\sum_{k=1}^{K}\eta_{k}^{\left(2\right)}. \end{array}$$ (43)

Here, $\lambda_{1}$ and $\lambda_{2}$ are positive penalty coefficients that regulate the impact of constraint violations. The penalty terms discourage infeasible solutions by reducing the reward when the SINR or secrecy capacity requirements are not met. This reward formulation enables the deep reinforcement learning (DRL) agent to balance system efficiency with constraint satisfaction. Furthermore, modifying only the reward structure allows both optimization objectives to be addressed within a unified learning framework.

The notations employed in the DRL-based spectral energy efficiency (SEE) optimization framework are summarized in Table 2.

**Table 2:** Notation for DRL-Based SEE Optimization.

Symbol	Description
$s_{t}$	System state at time step *t*
$a_{t}$	Action at time step *t*
$r_{t}$	Reward at time step *t*
𝒮	State space
𝒜	Action space
𝒫	State transition probability
ℛ	Reward function
γ	Discount factor
$\lambda_{1},\lambda_{2}$	Penalty coefficients in the reward function
$\eta_{k}^{\left(1\right)}$	Indicator of SINR constraint violation
$\eta_{k}^{\left(2\right)}$	Indicator of secrecy constraint violation
Q(s,a;θ)	Q-network with parameters θ
$\hat{Q}(s,a;\theta^{-})$	Target Q-network
𝒟	Experience replay buffer
Γ	Mini-batch size
*Ne*	Number of training episodes
*T*	Number of time steps per episode
$N_{L}$	Number of neural network layers
ϵ	Exploration parameter in ϵ-greedy policy

### 4.2 DQN-based algorithm for SEE optimization

In this subsection, a Deep Q-Network (DQN)-based algorithm is employed to solve the SEE optimization problem formulated as an MDP in Section 4.1. Due to the high dimensionality and non-convex nature of the problem, conventional optimization methods are difficult to apply. Therefore, DQN is adopted to learn an efficient resource allocation policy.

The DQN algorithm approximates the optimal action-value function $Q^{*}(s,a)$ using a deep neural network with parameters θ. Given a state $s_{t}\in 𝒮$, the agent selects an action $a_{t}\in 𝒜$ according to an ϵ-greedy policy, where exploration and exploitation are balanced.

At each time step, the agent observes a transition $\left(s_{t},a_{t},r_{t},s_{t+1}\right)$, where the reward $r_{t}$ is defined according to the SEE optimization objective described in Section 4.1.

Specifically, the reward is given as:

**Case 1 (Total SEE Maximization):**


$$\begin{array}{c} r_{t}^{\left(1\right)}={SEE}_{total}-\lambda_{1}\sum_{k=1}^{K}\eta_{k}^{\left(1\right)}-\lambda_{2}\sum_{k=1}^{K}\eta_{k}^{\left(2\right)} \end{array}$$ (44)

**Case 2 (Max–Min SEE):**


$$\begin{array}{c} r_{t}^{\left(2\right)}=\min_{k=1,\ldots ,K}\{SEE_{k}\}-\lambda_{1}\sum_{k=1}^{K}\eta_{k}^{\left(1\right)}-\lambda_{2}\sum_{k=1}^{K}\eta_{k}^{\left(2\right)} \end{array}$$ (45)

The experiences are stored in a replay buffer 𝒟, and mini-batches are sampled to train the network.

The target value for each transition $\left(s_{t},a_{t},r_{t},s_{t+1}\right)$ is computed as


$$\begin{array}{c} y_{t}^{\text{DQN}}=r_{t}+\gamma \max_{a^{\prime }}\hat{Q}(s_{t+1},a^{\prime };\theta^{-}) \end{array}$$ (46)

where $\hat{Q}(s,a;\theta^{-})$ is the target network, and γ∈[0,1] is the discount factor.

The loss function is defined as


$$\begin{array}{c} L(\theta )={𝔼}_{(s_{t},a_{t},r_{t},s_{t+1})\sim 𝒟}\left[{\left(y_{t}^{\text{DQN}}-Q(s_{t},a_{t};\theta )\right)}^{2}\right] \end{array}$$ (47)

The network parameters θ are updated via stochastic gradient descent, while the target network parameters $\theta^{-}$ are periodically updated to stabilize training.

The overall objective of the agent is to learn an optimal policy that jointly optimizes the power allocation vector α, IRS phase shift matrix Θ, and transmit powers $Q_{GS}$ and $Q_{J}$, so as to maximize the SEE while satisfying system constraints.

The detailed implementation of the proposed DQN-based SEE optimization is summarized in Algorithm 1.

**Algorithm 1 DQN-Based SEE Optimization Algorithm**

1:  Initialize Q-network Q(s,a;θ) with random weights θ

2:  Initialize target network $\hat{Q}(s,a;\theta^{-})$ with $\theta^{-}=\theta$

3:  Initialize replay buffer 𝒟 with capacity *N*

4:  **for** episode *e* = 1 to *Ne* **do**

5:   Initialize environment and obtain initial state *s*_1_

6:   **for** time step *t* = 1 to *T* **do**

7:    Select action $a_{t}$ using the ϵ-greedy policy

8:    Execute action $a_{t}$ to update α, Θ, $Q_{GS}$, and $Q_{J}$

9:    Observe next state $s_{t+1}$

10:    **if** Case 1 **then**

11:     Compute reward

$r_{t}={SEE}_{total}-\lambda_{1}\sum_{k=1}^{K}\eta_{k}^{\left(1\right)}-\lambda_{2}\sum_{k=1}^{K}\eta_{k}^{\left(2\right)}$

12:    **else**

13:     Compute reward

$r_{t}=\min_{k}\{SEE_{k}\}-\lambda_{1}\sum_{k=1}^{K}\eta_{k}^{\left(1\right)}-\lambda_{2}\sum_{k=1}^{K}\eta_{k}^{\left(2\right)}$

14:    **end if**

15:    Store transition $\left(s_{t},a_{t},r_{t},s_{t+1}\right)$ in 𝒟

16:    Sample a mini-batch from 𝒟

17:    **for** each sample $\left(s_{j},a_{j},r_{j},s_{j+1}\right)$ **do**

18:     Compute target

$y_{j}=\left\{ \begin{array}{cc} r_{j}, & \text{if terminal},\\ r_{j}+\gamma \max_{a^{\prime }}\hat{Q}(s_{j+1},a^{\prime };\theta^{-}), & \text{otherwise}. \end{array}\right.$

19:    **end for**

20:    Update θ by minimizing

$L=\frac{1}{\Gamma }\sum_{j}{\left(y_{j}-Q(s_{j},a_{j};\theta )\right)}^{2}$

21:    Every *C* steps, update target network: $\theta^{-}\leftarrow \theta$

22:   **end for**

23:  **end for**

### 4.3 Double DQN-based algorithm for SEE optimization

Building upon the DQN-based framework presented in the previous subsection, a Double Deep Q-Network (Double DQN)-based algorithm is adopted to further improve the stability and convergence of the learning process.

In comparison with conventional DQN, Double DQN can reduce overestimation bias by separating action selection from evaluation. Specifically, the action is selected using the main Q-network, while the Q-value is evaluated using the target network. The detailed implementation of the Double DQN-based SEE optimization is presented in Algorithm 2.

**Algorithm 2 Double DQN-Based SEE Optimization Algorithm**

1:  Initialize Q-network Q(s,a;θ) with random weights θ

2:  Initialize target network $\hat{Q}(s,a;\theta^{-})$ with $\theta^{-}=\theta$

3:  Initialize replay buffer 𝒟 with capacity *N*

4:  **for** episode *e* = 1 to *Ne* **do**

5:   Initialize environment and obtain initial state *s*_1_

6:   **for** time step *t* = 1 to *T* **do**

7:    Select action $a_{t}$ using ϵ-greedy policy

8:    Execute action $a_{t}$ to update $\alpha ,\Theta ,Q_{GS},Q_{J}$

9:    Observe next state $s_{t+1}$

10:    **if** Case 1 **then**

11:     Compute reward

$r_{t}={SEE}_{total}-\lambda_{1}\sum_{k=1}^{K}\eta_{k}^{\left(1\right)}-\lambda_{2}\sum_{k=1}^{K}\eta_{k}^{\left(2\right)}$

12:    **else**

13:     Compute reward

$r_{t}=\min_{k}\{SEE_{k}\}-\lambda_{1}\sum_{k=1}^{K}\eta_{k}^{\left(1\right)}-\lambda_{2}\sum_{k=1}^{K}\eta_{k}^{\left(2\right)}$

14:    **end if**

15:    Store $\left(s_{t},a_{t},r_{t},s_{t+1}\right)$ in 𝒟

16:    Sample mini-batch from 𝒟

17:    **for** each sample $\left(s_{j},a_{j},r_{j},s_{j+1}\right)$ **do**

18:     Select action:

$a^{*}=\text{arg}\max_{a^{\prime }}Q(s_{j+1},a^{\prime };\theta )$

19:     Compute target:

$y_{j}=\left\{ \begin{array}{cc} r_{j}, & \text{if terminal},\\ r_{j}+\gamma \hat{Q}(s_{j+1},a^{*};\theta^{-}), & \text{otherwise} \end{array}\right.$

20:    **end for**

21:    Update θ by minimizing

$L=\frac{1}{\Gamma }\sum_{j}{\left(y_{j}-Q(s_{j},a_{j};\theta )\right)}^{2}$

22:    Every *C* steps: $\theta^{-}\leftarrow \theta$

23:   **end for**

24:  **end for**

The computational complexity of both DQN and Double DQN algorithms is approximately $𝒪(Ne\cdot T\cdot \Gamma \cdot N_{L})$, where *Ne* is the number of episodes, *T* is the number of time steps, Γ is the mini-batch size, and $N_{L}$ is the number of neural network layers. Compared with DQN, the Double DQN algorithm introduces negligible additional computational complexity while significantly improving training stability by reducing overestimation bias.

The ϵ-greedy strategy balances exploration and exploitation, with ϵ gradually decreasing over training. Moreover, the experience replay mechanism enhances learning efficiency by breaking temporal correlations among samples.

Furthermore, the proposed Double DQN framework follows an offline training and online execution paradigm, which is well-suited for real-world deployment. Specifically, the agent is trained offline over *E* episodes using a simulated THz-Rician channel environment that statistically reflects the actual propagation conditions. The training phase, which has complexity $𝒪(Ne\cdot T\cdot \Gamma \cdot N_{L})$, is performed once Prior to deployment, it imposes no real-time burden on the network. Once training is complete, the online execution phase requires only a single forward pass through the trained Q-network to determine the optimal action $a_{t}$ given the observed state $s_{t}$. This inference operation has a fixed computational complexity of $𝒪(N_{L})$, and can be executed in a few milliseconds on modern edge computing hardware or dedicated AI inference accelerators. This latency is well within the channel coherence time of the quasi-static THz-Rician fading model considered, making the approach suitable for real-time resource management.

## 5 Experimental results and discussion

In this section, we evaluate the performance of the proposed IRS-assisted NOMA framework in terms of secrecy energy efficiency (SEE). Two optimization strategies are considered: total SEE maximization and max-min SEE optimization. Furthermore, the user fairness index is investigated to highlight the trade-off between efficiency and fairness.

All simulations are implemented in MATLAB and Google Colab. The default system parameters are listed in Table 3. To provide clear insights while maintaining analytical generality, simulations are performed in a two-user scenario (*U*_1_ and *U*_2_), whereas the theoretical formulation is derived for a general system with *K* users.

**Table 3:** Simulation Parameters.

Parameter	Value
Transmit power of GS, $Q_{GS}$	35 dBm
Transmit power of jammer, $Q_{J}$	30 dBm
Carrier frequency, *f*	1 THz
System bandwidth, *B*	10 MHz
Atmospheric absorption coefficient, $k_{f}$	0.5
Noise power at legitimate users, $\delta_{{LU}_{k}}^{2}$	−90 dBm
Noise power at eavesdropper, $\delta_{E}^{2}$	−90 dBm
Number of IRS elements, *M*	64
Power amplifier efficiency at GS, $\beta_{PAE}^{GS}$	0.5
Power amplifier efficiency at jammer, $\beta_{PAE}^{jammer}$	0.4
Circuit power at GS, $P_{c}^{GS}$	1 W
Circuit power per user, $P_{c}^{U}$	0.1 W
Circuit power per IRS element, $P_{c}^{IRS,elem}$	5 mW
IRS controller power, $P_{c}^{IRS,ctrl}$	0.1 W
Distance GS–IRS, $d_{GS,IRS}$	10 m
Distance IRS–*U*_1_, $d_{IRS,U_{1}}$	15 m
Distance IRS–*U*_2_, $d_{IRS,U_{2}}$	18 m
Distance IRS–E, $d_{IRS,E}$	25 m
Distance GS–*U*_1_, $d_{GS,U_{1}}$	18 m
Distance GS–*U*_2_, $d_{GS,U_{2}}$	22 m
Distance GS–E, $d_{GS,E}$	28 m
Distance jammer–E, $d_{J,E}$	20 m

As a benchmark, an OMA-based scheme (FDMA) is considered, in which the system bandwidth is divided among users via source allocation factors $\beta_{1}$ and $\beta_{2}$. For a fair comparison, the same optimization framework is applied to both the proposed NOMA scheme and the OMA baseline. Note that among OMA schemes, FDMA is selected as the most suitable representative for this system because it maintains a fixed IRS phase-shift matrix Θ across users and is naturally compatible with the wideband THz channel model. In comparison, TDMA would require an IRS reconfiguration at every time slot and reduce each user’s effective throughput by a factor of $\frac{1}{K}$, resulting in lower SEE—suggesting that NOMA’s advantage over TDMA would be even more pronounced than the NOMA-vs-FDMA gap shown in our results.

### 5.1 Sum SEE performance analysis (Case 1)

**Fig 2:**
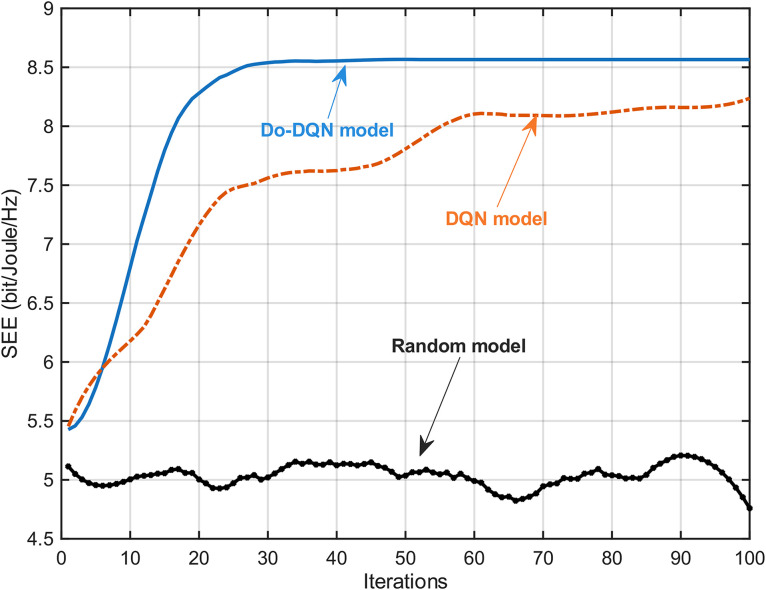
Convergence Behavior secrecy energy efficiency (SEE) versus iterations for different algorithms.

Fig 2 illustrates the convergence behavior of secrecy energy efficiency (SEE) with respect to the number of iterations for three approaches: our proposed Double DQN model (or Do-DQN model), the DQN model, and the random model, in the considered NOMA-IRS system with an eavesdropper and a friendly jammer. Our proposed Double DQN model achieves the fastest convergence among the three methods. The SEE value increases rapidly during the initial iterations, particularly from iteration 5–20, then gradually stabilizes at approximately 8.5 bits/Joule/Hz. This is the highest value of the objective function compared with the DQN and Random methods. This result demonstrates that the Double DQN model can learn an efficient policy to optimize the system’s SEE. In contrast, the DQN model also shows an increasing trend in SEE with increasing iterations is slower than that of the Double DQN model. And the DQN algorithm achieves a lower SEE value of approximately 8.1–8.2 bit/Joule/Hz. Meanwhile, the random method maintained significantly lower SEE; the achieved SEE values ranged from 4.8 to 5.2 bits/Joule/Hz and showed no clear convergence trend. The main reason is that the random strategy did not leverage system state information to improve decision-making. Overall, the results in Fig 2 demonstrate that the Double DQN-based approach significantly outperforms both the DQN and random models in terms of convergence speed, stability, and SEE performance, highlighting the effectiveness of the proposed learning-based optimization scheme for the considered NOMA-IRS system with a friendly jammer.

It is evident that the Double DQN algorithm converges faster and achieves the highest SEE performance among all considered methods. Hence, in the subsequent experiments, only the trained Double DQN model is employed for performance evaluation to ensure both efficiency and stability.

**Fig 3:**
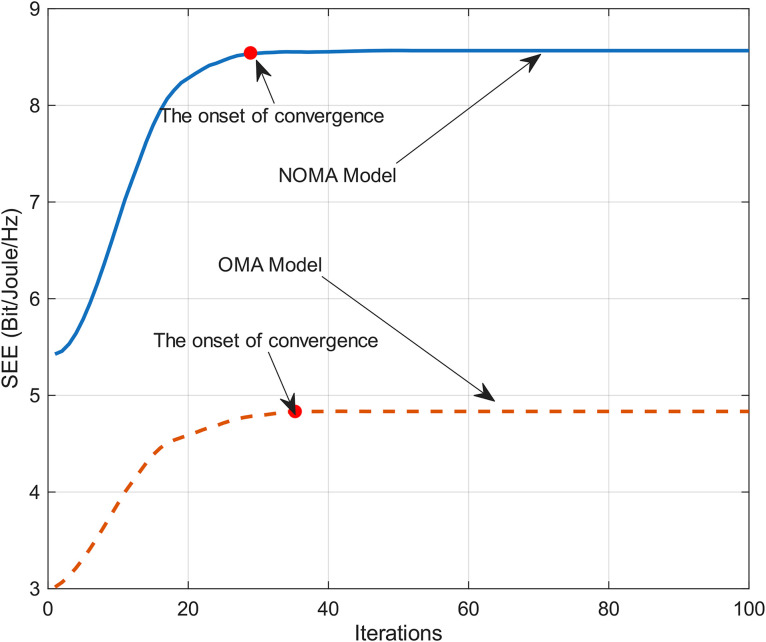
Convergence behavior of SEE for NOMA and OMA schemes under the Double DQN algorithm.

Fig 3 presents the convergence lines of the SEE for both IRS-assisted NOMA (solid line) and IRS-assisted OMA (dashed line) systems under the Double DQN model. Both systems gradually improve their SEE values as the number of iterations increases and eventually converge to stable performance levels. From Fig 3, we can see that the NOMA system achieved higher SEE than the OMA system in all iterations. Specifically, NOMA achieved the highest and most stable SEE value of 8.5 bits/Joule/Hz, while OMA reached only about 5 bits/Joule/Hz. Furthermore, the NOMA model converged faster, reaching stable performance after approximately 25 iterations, while the OMA method converged more slowly and stabilized at a lower SEE.

**Fig 4:**
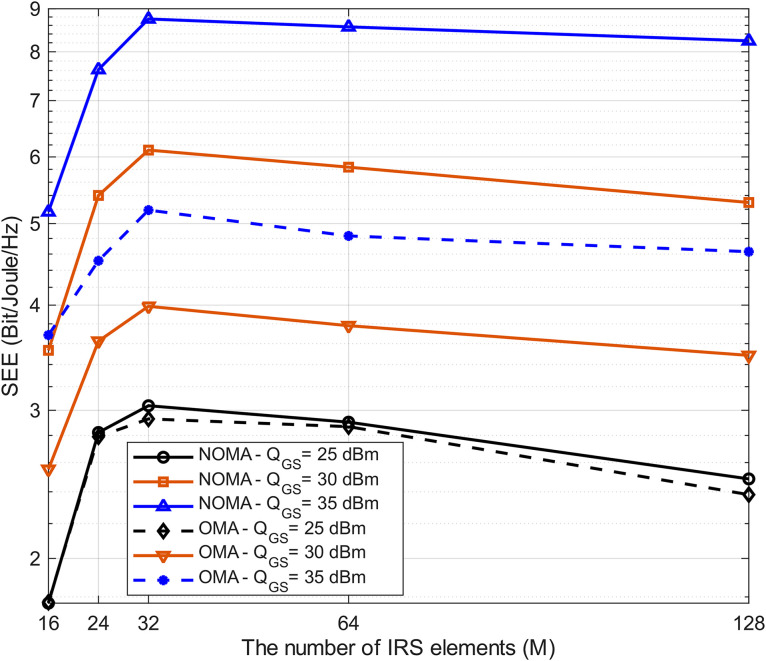
Effects of the number of IRS elements on secrecy energy efficiency (SEE).

Fig 4 shows the effects of the number of IRS reflecting elements, M, on the sum of secrecy energy efficiency (SEE) at different base station transmit power levels: $P_{S}$=25 dBm, $P_{S}$=30 dBm, and $P_{S}$=35 dBm. The SEE initially increases as the number of IRS elements grows from M = 16 to M = 32. However, as the number of reflecting elements increases beyond M = 32, the sum of SEE gradually decreases. This result can be explained as follows. A larger number of IRS elements improves the received signal power, but also increases circuit power consumption. As a result, the gain in energy efficiency becomes marginal and eventually decreases when hardware power consumption dominates the power of the system. Fig 4 also shows that higher base-station transmit power leads to better SEE performance. Specifically, the sum of SEE achieved with $P_{S}$ = 35 dBm is consistently higher than that with $P_{S}$ = 30 dBm and $P_{S}$ = 25 dBm in all the values considered of M. Nevertheless, excessive transmit power may also increase overall energy consumption, which helps explain the relatively small improvement in SEE as M increases. Overall, the results indicate that there is an optimal number of IRS elements (around M = 32 in this scenario) that maximizes SEE for the considered NOMA-IRS system. Further increasing the IRS size does not improve SEE due to additional hardware power consumption. Finally, from Fig 4, we see that the total SEE of our proposed NOMA (solid lines) system is higher than that of the OMA (dashed lines) system across the cases considered.

**Fig 5:**
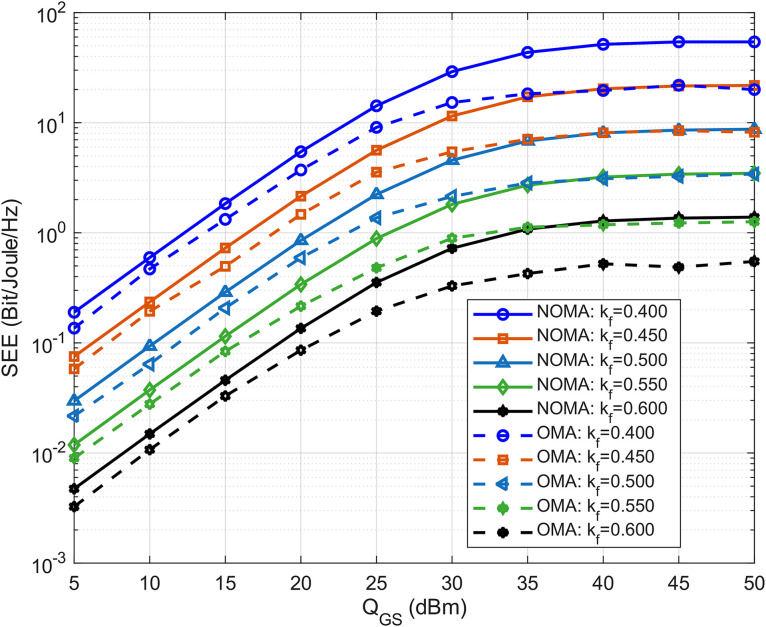
Effects of the atmospheric absorption coefficients $k_{f}$ on secrecy energy efficiency (SEE).

Fig 5 shows the variation of total secure energy efficiency (SEE) with transmit power $Q_{GS}$ for different atmospheric absorption coefficients $k_{f}$ in the considered THz communication channel. The transmit power ranges from 5 dBm to 50 dBm. In the simulation, the absorption coefficient $k_{f}$ varies from 0.4 to 0.60, to represent various atmospheric absorption conditions in THz wireless environments. The total SEE increases monotonically with transmit power for all values of $k_{f}$. As the transmit power increases from 5 dBm to about 35 dBm, the total SEE improves. This is due to the enhanced received signal strength at the legitimate user, which increases the system’s achievable security capacity and overall energy-security efficiency. However, at about 40 dBm, SEE growth slows and approaches saturation. The figure also shows that the atmospheric absorption coefficient $k_{f}$ significantly affects the system’s performance. Lower $k_{f}$ values give much higher total SEE. The system achieves the highest SEE at $k_{f}=0.4$ for all transmit power levels. In contrast, $k_{f}=0.60$ yields the lowest SEE. This outcome is typical in THz communication. Higher absorption coefficients result in greater atmospheric signal attenuation, weakening the received signal power at valid receivers and reducing the achieved security rate and overall energy efficiency. These results show that both transmit power and the atmospheric absorption properties of the THz channel are critical for total SEE performance. Finally, the Fig 5 shows that the total SEE of our proposed NOMA (solid lines) system is higher than that of the OMA (dashed lines) system across the cases considered.

**Fig 6:**
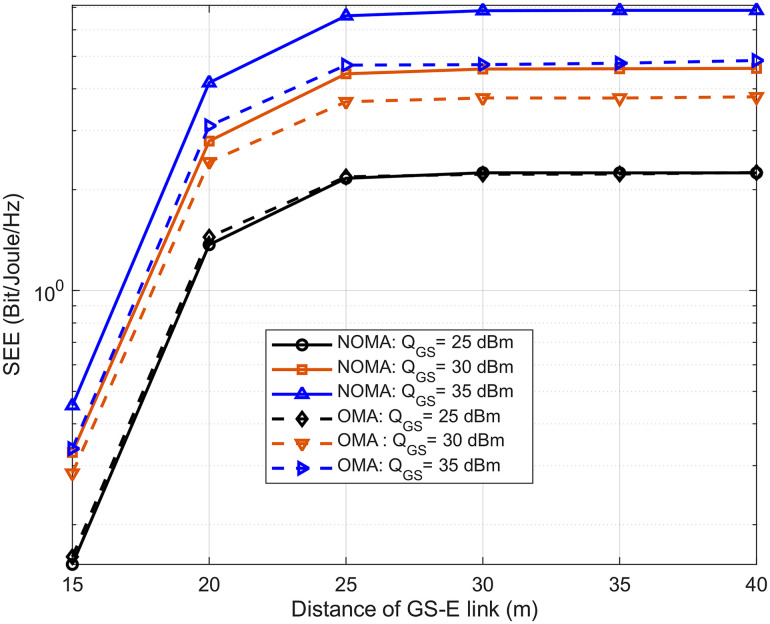
Effects of distance E-GBS link on secrecy energy efficiency (SEE).

Fig 6 illustrates the effect of the distance between the eavesdropper and the ground base station (GS) on the total secrecy energy efficiency (SEE) across different transmit power levels $P_{s}$. As the Eve–GBS link distance increases from 10 m to 40 m, the SEE improves significantly, particularly between 10 m and 20 m. For eavesdroppers close to the base station (10 m), the SEE remains low for all transmit power levels because the strong received signal at the eavesdropper reduces the secrecy rate and degrades overall SEE. When the distance grows beyond 25 m, SEE stabilizes, indicating that further increases in distance yield negligible improvement. The figure also demonstrates that higher transmit power results in better SEE, with $P_{s}=35$ dBm performing best, followed by $P_{s}=30$ dBm and $P_{s}=25$ dBm, as increased power benefits legitimate users’ signal quality. Overall, the results confirm that greater separation between the eavesdropper and the base station and higher transmit power boost SEE in the NOMA-IRS system. Lastly, the NOMA scheme (solid lines) consistently achieves a higher SEE than the OMA scheme (dashed lines) across all conditions considered. This performance gain is due to the superior spectral efficiency and more flexible resource allocation of NOMA, which enables more efficient utilization of transmit power than OMA.

Although the results in Section 5.1 highlight the benefit of maximizing total SEE, such a strategy does not always guarantee a fair performance across users. In particular, users with weaker channels may suffer from significantly lower SEE. To alleviate this issue, we next consider a max–min SEE formulation that explicitly targets the worst-case user.

### 5.2 Max–Min SEE Performance Analysis (Case 2)

**Fig 7:**
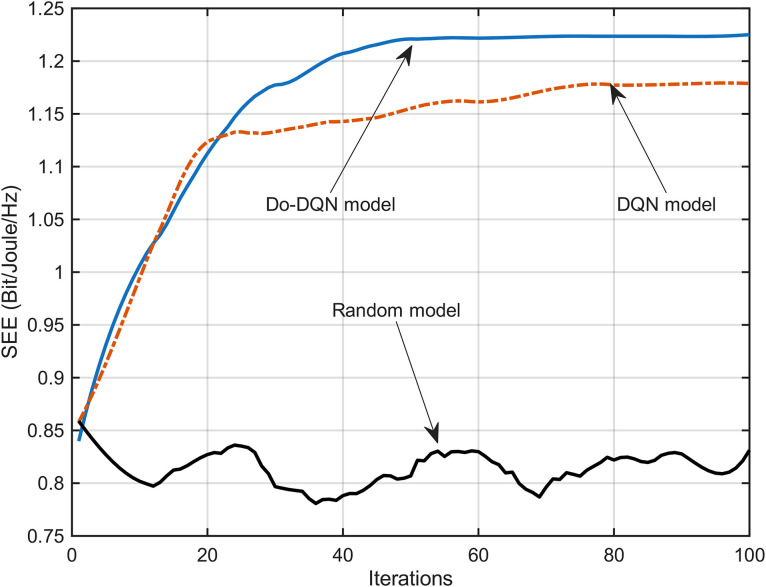
Convergence Behavior secrecy energy efficiency (SEE) versus iterations for different algorithms (Max-min Case).

In this subsection, we examine the SEE performance of the proposed IRS-assisted NOMA system under the max–min optimization framework (Case 2), which aims to improve the worst-case user’s performance by maximizing the minimum SEE across all users (Fig 7).

**Fig 8:**
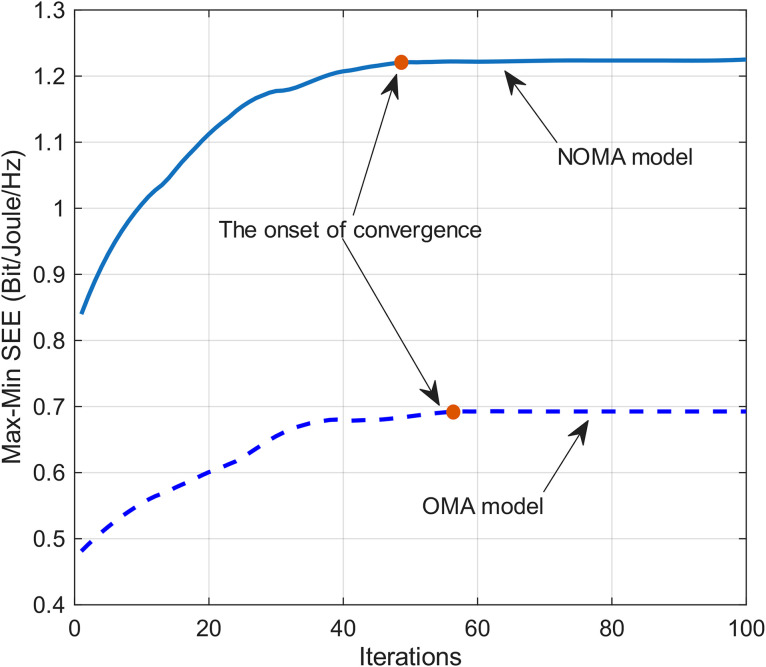
Comparison of the convergence Behavior SEE between our proposed NOMA and OMA models (Max-min Case).

Fig 7 and 8 shows the convergence behavior of the considered algorithms under the max–min SEE criterion. Unlike the sum-SEE case, where the objective function is evaluated based on the total SEE across all users, the max–min formulation focuses only on the worst-case user, i.e., the user with the minimum SEE. Therefore, the converged SEE value in this figure is naturally much lower than that obtained in the sum-SEE case, since it only reflects the performance of the weakest user rather than the aggregate system performance. From the figure, it can be observed that the learning-based algorithms still exhibit clear convergence behavior under the max–min optimization framework. In particular, the proposed Double DQN model converges faster and achieves a higher minimum SEE than the conventional DQN model, whereas the random model fluctuates at a much lower level and shows no clear convergence trend. This result confirms that our proposed algorithm effectively solves the fairness-oriented max—min SEE optimization problem and maintains stable learning performance even under a more stringent objective function.

The results above confirm that the proposed Do-DQN-based algorithm can achieve stable convergence within the max–min SEE optimization framework. After validating the convergence of the learning process, the subsequent simulations evaluate the system performance using the sum SEE metric to further investigate the overall efficiency of the considered NOMA–IRS system.

**Fig 9:**
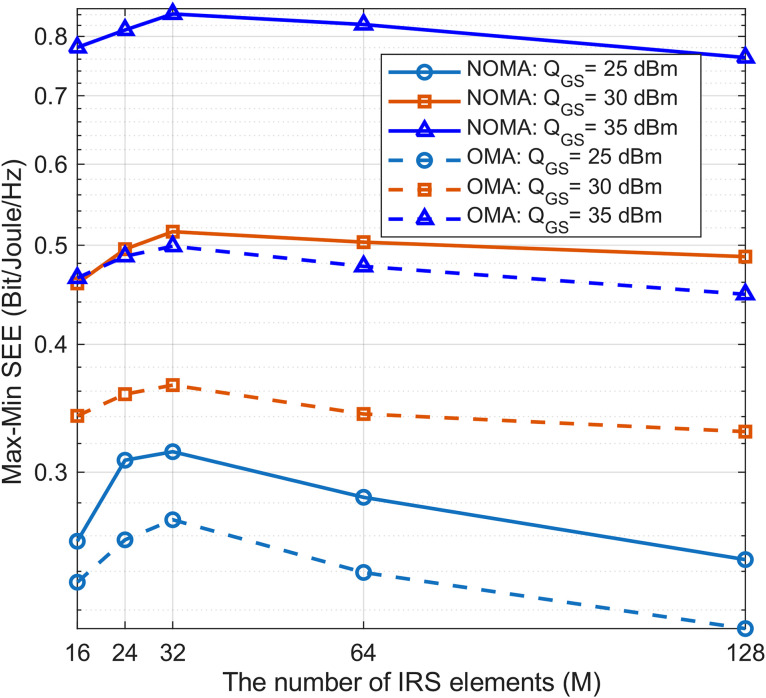
Effects of M on Max-Min(SEE).

Fig 9 illustrates the impact of the number of IRS reflecting elements *M* on the secrecy energy efficiency (SEE) under different transmit power levels $Q_{GS}$ for both NOMA and OMA schemes. It is worth noting that the learning model is trained using the max–min SEE criterion to guarantee fairness among users, while the performance shown in this figure is evaluated as the sum SEE across all users after training. From Fig 6, we can observe that the SEE first increases as the number of IRS elements grows from *M* = 16 to *M* = 32. However, as the number of IRS elements increases beyond *M* = 32, the SEE gradually decreases. This phenomenon can be attributed to additional power consumption in the circuit due to the IRS elements. Although a larger IRS can further enhance signal gain, the increased hardware power consumption reduces the system’s overall energy efficiency. In addition, the figure shows that higher transmit power leads to better SEE performance across all values of *M*. Specifically, the case with $P_{s}=35$ dBm consistently achieves the highest SEE, followed by $P_{s}=30$ dBm and $P_{s}=25$ dBm. This is because higher transmit power increases the achievable secrecy capacity of legitimate users, thereby increasing the SEE. Furthermore, the NOMA scheme consistently outperforms the OMA scheme across all configurations, demonstrating its superiority in improving the worst-user performance. This confirms that NOMA is more effective at achieving fairness-oriented optimization than conventional OMA.

**Fig 10:**
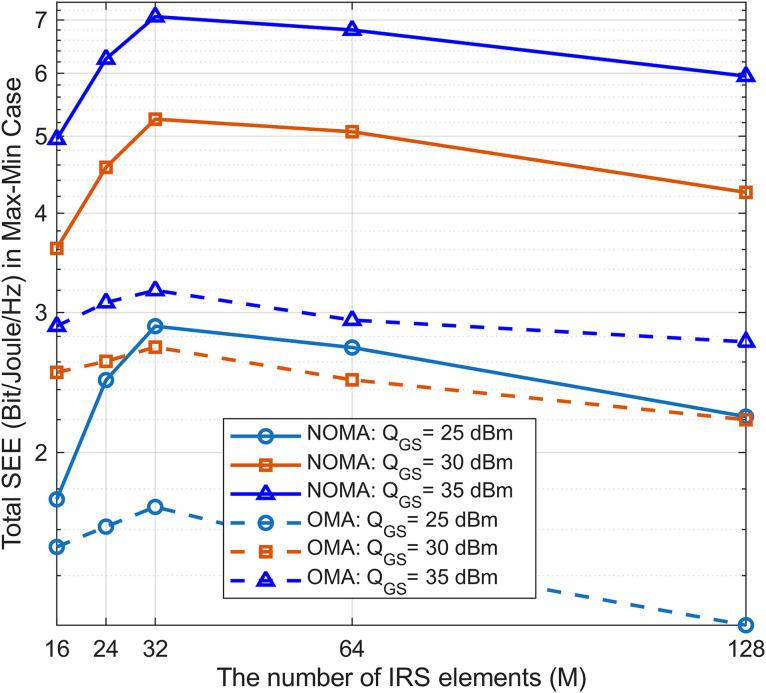
Effects of M on total SEE with Max-min Case.

**Fig 11:**
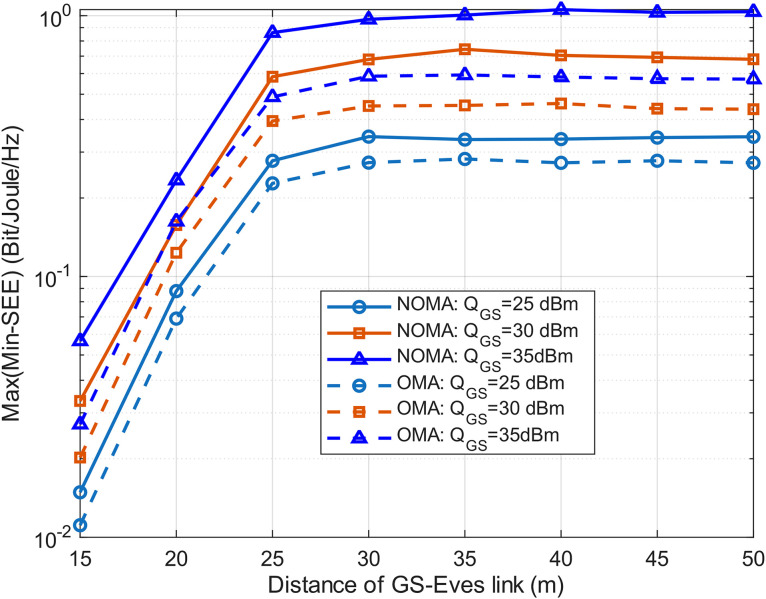
Effects of distance E-GBS link on Max-Min(SEE).

Fig 10 presents the corresponding total SEE achieved under the optimal policy obtained from the max–min SEE optimization problem for both NOMA and OMA schemes. It can be observed that the total SEE follows a similar trend: it increases with *M* up to a certain point, then decreases due to the rising IRS power consumption. However, compared to the total SEE obtained from the sum SEE maximization (Case 1), the values in Fig 11 are relatively lower. This is because the max–min optimization prioritizes worst-case user performance, inevitably sacrificing some of the overall system efficiency. However, these results show that our proposed IRS-assisted NOMA system still achieves a significantly higher total SEE than the OMA scheme, meaning that NOMA maintains its efficiency advantage even during the optimization process aimed at equity.

Fig 11 illustrates the impact of the distance between the eavesdropper and the ground base station (Eve–GS link) on the secrecy energy efficiency (SEE) under different transmit power levels $P_{s}$. It should be noted that the learning model is trained using the max–min SEE criterion to ensure fairness among users, while the performance shown in this figure is evaluated based on the sum SEE after training. From the figure, it can be observed that the SEE increases significantly as the distance between the E and GS increases. When the E is located close to the base station (e.g., 10 m), the SEE remains low. And, the distance increases from 10 m to approximately 20 m, the SEE improves rapidly for all considered transmit power levels. When the eavesdropper is located further away from the base station (beyond 25 m), the SEE gradually approaches a stable region. In addition, it is clear that higher transmit power levels consistently yield better SEE performance. Specifically, the case with $P_{s}=35$ dBm provides the highest SEE, followed by $P_{s}=30$ dBm and $P_{s}=25$ dBm. This is because a higher transmit power increases the received signal strength at legitimate users, thereby enhancing the achievable secrecy rate and increasing the system’s overall secrecy energy efficiency. Finally, Fig 11 also shows that the NOMA system achieves better minimum SEE efficiency than the OMA system at different transmit power levels.

**Fig 12:**
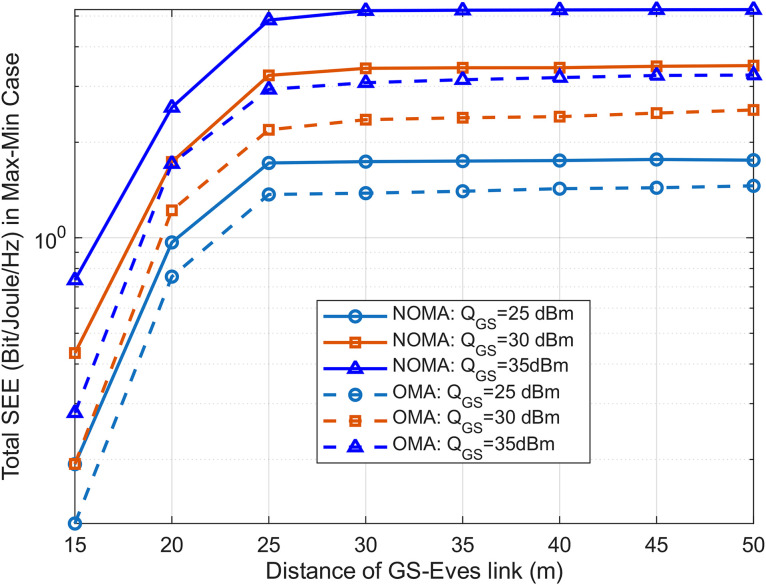
Effects of distance E-GBS link on total SEE with Max-min Case.

Fig 12 illustrates the impact of the GS–eavesdropper (GS–E) distance on the total SEE under the max–min SEE optimization strategy. It can be observed that the total SEE increases significantly as the GS–E distance increases from 15 m to approximately 25 m, then gradually saturates at larger distances. Moreover, the proposed IRS-assisted NOMA scheme consistently outperforms the OMA scheme across all considered transmit power levels. It is also observed that the improvement in total SEE diminishes with increasing distance. These results confirm that, even under the fairness-oriented max–min SEE optimization, the system can still achieve considerable total SEE gains, especially when the eavesdropper is located farther from the transmitter.

**Fig 13:**
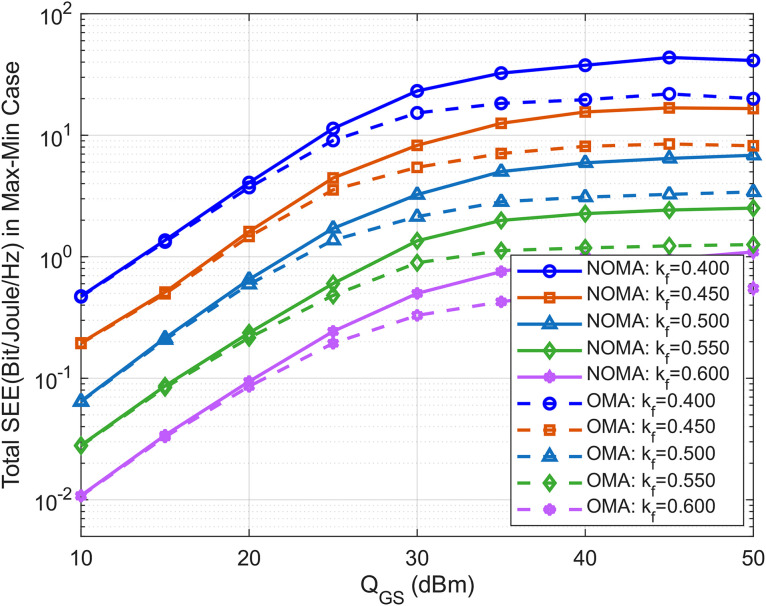
Effects of the atmospheric absorption coefficients $k_{f}$ on total (SEE) with Max-Min case.

Fig 13 illustrates the impact of the atmospheric absorption coefficient $k_{f}$ on the total SEE under the max–min SEE optimization framework for both NOMA and OMA systems. It can be observed that the total SEE increases monotonically with the transmit power $Q_{GS}$ for all considered values of $k_{f}$. This is because higher transmit power increases the received signal strength for legitimate users, thereby improving secrecy capacity and, consequently, the overall SEE. However, the performance is significantly affected by the atmospheric absorption coefficient. Specifically, as $k_{f}$ increases from 0.4 to 0.6, the total SEE degrades considerably for both NOMA and OMA schemes. Furthermore, the proposed NOMA model based on IRS achieves significantly better SEE performance than the OMA model across all values of $k_{f}$ and $Q_{GS}$. More specifically, the performance gap becomes more pronounced at lower absorption levels (e.g., $k_{f}=0.4$), where channel conditions are more favorable, enabling NOMA to better exploit multiplexing in the power domain. Meanwhile, under severe absorption conditions (e.g., $k_{f}=0.6$), the performance of both options decreases, and the gap between NOMA and OMA becomes less significant. Finally, although maximum-minimum SEE optimization prioritizes fairness among users, the total SEE still exhibits a clear performance trend with both transmit power and channel conditions, demonstrating the robustness of the proposed framework across various THz transmission environments.

### 5.3 Trade-off analysis between SEE and fairness

**Fig 14:**
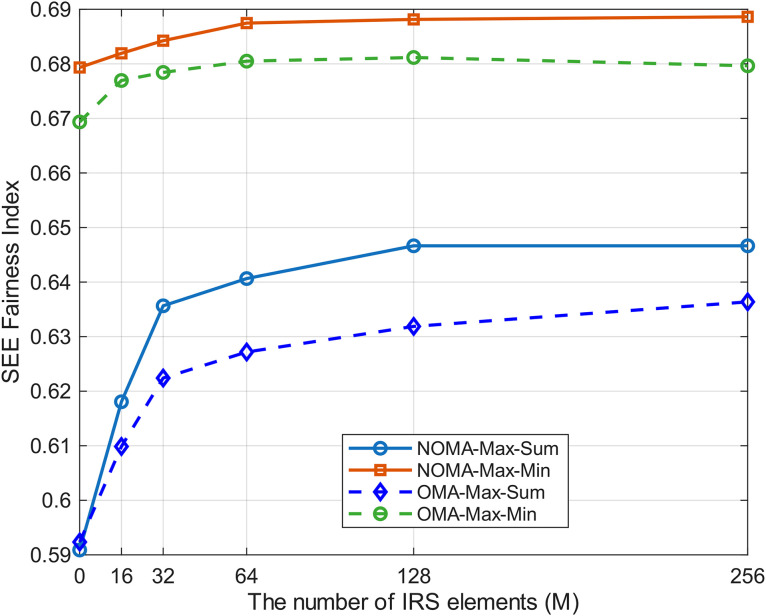
User Fairness Index between Max-Sum SEE and Max-Min SEE cases versus M-IRS elements.

To further investigate the fairness performance of the considered system, we evaluated Jain’s fairness index among users under both the max–sum SEE and max–min SEE optimization schemes. Fig 14 illustrates the fairness index for both NOMA and OMA systems in both cases (case 1 and case 2) versus the number of IRS elements. It can be observed that the max–min optimization consistently achieves a higher fairness index compared to the max–sum SEE scheme. This is expected because the max–min formulation explicitly maximizes the SEE of the worst user, thereby balancing the performance among users. Furthermore, Fig 14 shows that the User Fairness Index of the OMA system is similar to that of the NOMA system. As *M* increases from small values, the fairness index increases and then stabilizes. However, OMA consistently exhibits lower levels of fairness than NOMA in both scenarios. This result may be due to OMA’s fixed resource allocation, which limits its ability to balance user performance. These findings suggest that NOMA uses IRS more effectively to enhance SEE balance among users.

**Fig 15:**
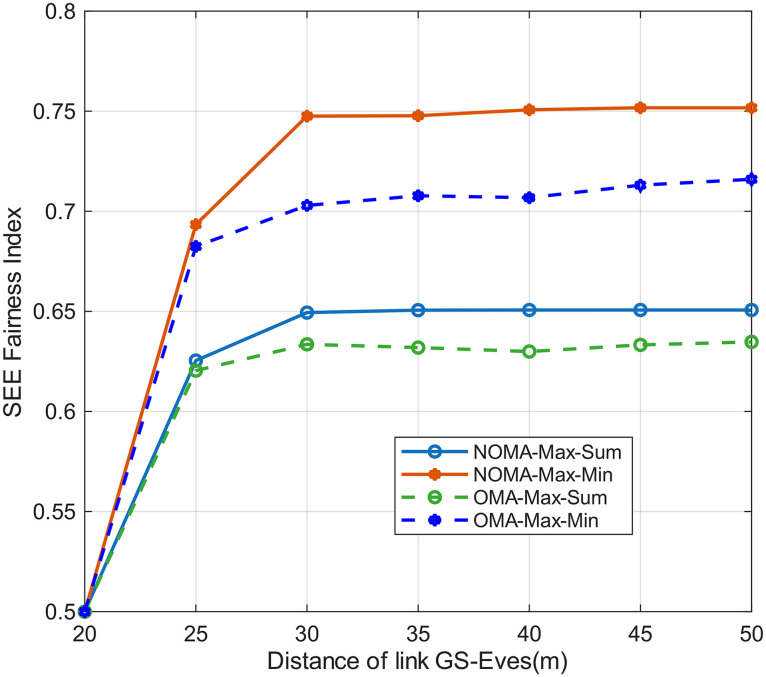
Effects of Distance GS-E link on Fairness Index between Max-Sum SEE and Max-Min SEE cases.

Similarly, Fig 15 presents the fairness index with respect to the distance between the ground station and the eavesdropper. The results again show that the max–min scheme significantly improves the system’s fairness performance. Although the max–min approach may yield a slightly lower sum SEE than the max–sum scheme, it effectively enhances fairness among users by preventing the performance of weaker users from being excessively degraded. The OMA results follow a similar trend. The fairness index increases quickly as the GS–E distance grows from small values, and then changes only slightly when the distance becomes larger. Even so, OMA still offers lower fairness than NOMA in both cases. The gap is more pronounced in the max–min setting, where NOMA is more effective at improving the weakest user’s performance. These observations indicate that, although OMA benefits from better channel conditions, it is less flexible in balancing user performance. On the other hand, NOMA achieves a more even distribution of SEE among users.

Overall, these results confirm that the proposed max-min SEE optimization framework provides a better balance between system efficiency and user fairness in the considered NOMA–IRS secure communication system.

## 6 Conclusion

In this paper, we investigated the IRS-assisted NOMA network model in the presence of an eavesdropper and a friendly jammer under THz communication channels. Two optimization objectives were considered: maximizing total SEE and the max–min SEE design, which aims to improve the performance of the worst-case user. To address the high non-convexity and the constraint system variables, a Markov Decision Process (MDP) and deep reinforcement learning techniques, including Deep Q-Network (DQN) and Double DQN, were employed. The simulation results demonstrated that Double DQN converges faster and achieves better SEE performance than conventional DQN and random strategies. Furthermore, to highlight the SEE of our proposed NOMA system across various system configurations, the OMA system was used as a benchmark. Although the max–min SEE formulation reduces total SEE relative to sum-SEE maximization, it significantly improves the performance of the weakest user, resulting in a more balanced system. The impacts of important system parameters, such as the number of IRS elements, the GS–eavesdropper distance, and the THz absorption coefficient, were also analyzed, showing their critical role in SEE performance. Overall, these results confirm that the integration of NOMA, IRS, and DRL provides an effective and energy-efficient framework for enhancing physical layer security while ensuring user fairness. The proposed approach offers a promising solution for sustainable, green 6G wireless communication systems operating in dynamic and complex environments.

## Supporting information

S1 Appendix A Proof of Proposition 1. (PDF)
