# Correction: Double DQN-based secrecy energy efficiency and fairness performance in IRS-assisted NOMA systems with friendly jamming

**DOI:** 10.1371/journal.pone.0356627

**Published:** 2026-08-18

**Authors:** Hong Nguyen-Thi, Ngan Chu-Thi, Sang Quang-Nguyen, Dung Tran-Tien, Anh Le-Thi

There is an error in affiliation 2 for authors Ngan Chu-Thi, Dung Tran-Tien and Anh Le-Thi. The correct affiliation 2 is: School of Information and Communication Technology, Hanoi University of Industry, Hanoi, Vietnam.
