# Supplementary material for: Double DQN-based secrecy energy efficiency and fairness performance in IRS-assisted NOMA systems with friendly jamming
Source: PLoS One. 2026 Jul 1;21(7):e0352324. doi: 10.1371/journal.pone.0352324 (PMC13322567; doi:10.1371/journal.pone.0352324)
Supplement: S1 Appendix A — (PDF) [file pone.0352324.s001.pdf]

Appendix A: Proof of Proposition 1

We first consider the SINR constraint in (32e). This implicitly imposes a lower bound on the power allocation coefficient α_k, which depends on the SINR threshold, channel gain, and the power allocation of the other users. By substituting Eq.(12) into Eq.32e, we have

$$\frac{\alpha_k Q_{\mathrm{GS}} \left\| h_k^{\mathrm{GS}} + \mathbf{g}_{\mathrm{LU}_k}^H \mathbf{\Theta} \mathbf{g}_{SR} \right\|^2}{Q_{\mathrm{GS}} \left(\sum\limits_{j=1}^{k-1} \alpha_j + \xi \sum\limits_{j=k+1}^{K} \alpha_j \right) \left\| h_k^{\mathrm{GS}} + \mathbf{g}_{\mathrm{LU}_k}^H \mathbf{\Theta} \mathbf{g}_{SR} \right\|^2 + \delta_{\mathrm{LU}_k}^2} \geq \gamma_k^{th} \tag{1}$$

Thus, the feasible value of α_k must satisfy

$$\alpha_k \geq \gamma_k^{th} Q_{\mathrm{GS}} \left(\sum_{j=1}^{k-1} \alpha_j + \xi \sum_{j=k+1}^{K} \alpha_j \right) + \frac{\gamma_k^{th} \delta_{\mathrm{LU}_k}^2}{Q_{\mathrm{GS}} \left\| h_k^{\mathrm{GS}} + \mathbf{g}_{\mathrm{LU}_k}^H \mathbf{\Theta} \mathbf{g}_{SR} \right\|^2} \tag{2}$$

Since this lower bound still depends on the other users' power allocations, it is not a fixed bound. Therefore, in the optimization problem, constraint (33e) is retained in its original form. For initialization purposes, a simplified lower bound can be considered by neglecting the interference term, which yields

$$\alpha_k^{ini} = \frac{\gamma_k^{th} \delta_{\mathrm{LU}_k}^2}{Q_{\mathrm{GS}} \left\| h_k^{\mathrm{GS}} + \mathbf{g}_{\mathrm{LU}_k}^H \mathbf{\Theta} \mathbf{g}_{SR} \right\|^2}. \tag{3}$$

Moreover, we will consider the constraint in Eq.32f. To simplify the secrecy-capacity constraint, let $\tau_0 \triangleq 2^{2^{\frac{SC_0^{th}}{B}}}$, and define as follows

$$A_k \triangleq \sum_{j=1}^{k-1} \alpha_j + \xi \sum_{j=k+1}^{K} \alpha_j, \tag{4}$$

$$C_k \triangleq \frac{\delta_{\mathrm{LU}_k}^2}{Q_{\mathrm{GS}} \left\| h_k^{\mathrm{GS}} + \mathbf{g}_{\mathrm{LU}_k}^H \mathbf{\Theta} \mathbf{g}_{SR} \right\|^2}, \tag{5}$$

$$D_k \triangleq \frac{Q_J \left| h_E^J \right|^2}{Q_{\mathrm{GS}} \left\| h_E^{\mathrm{GS}} + \mathbf{g}_E^H \mathbf{\Theta} \mathbf{g}_{SR} \right\|^2} + \frac{\delta_E^2}{Q_{\mathrm{GS}} \left\| h_E^{\mathrm{GS}} + \mathbf{g}_E^H \mathbf{\Theta} \mathbf{g}_{SR} \right\|^2}. \tag{6}$$

Then, the secrecy-capacity constraint $SC_k^{\mathrm{IRS}} \geq SC_0^{\mathrm{th}}$ can be rewritten as

$$\begin{array}{l} SC_k^{\mathrm{IRS}} = \left[C_{\mathrm{LU}_k}^{[k]} - C_E^{[k]} \right]^+ \\ \frac{1+\frac{\alpha_k}{A_k+C_k}}{1+\frac{\alpha_k}{A_k+D_k}} \geq \tau_0 \end{array}, \tag{7}$$

which yields

$$\alpha_k [D_k - \tau_0 C_k - (\tau_0 - 1) A_k] \geq (\tau_0 - 1)(A_k + C_k)(A_k + D_k). \tag{8}$$

Hence, the secrecy-capacity requirement implicitly imposes the following lower bound on α_k:

$$\alpha_k \geq \frac{(\tau_0 - 1)(A_k + C_k)(A_k + D_k)}{D_k - \tau_0 C_k - (\tau_0 - 1)A_k}, \tag{9}$$

provided that

$$D_k - \tau_0 C_k - (\tau_0 - 1)A_k > 0. \tag{10}$$

Similar to the SINR constraint, this lower bound also depends on A_k, and hence on other users' power allocation.

Therefore, both constraints jointly impose implicit and coupled lower bounds on α_k, which completes the proof.
